# Role of the P-Type ATPases, ATP7A and ATP7B in brain copper homeostasis

**DOI:** 10.3389/fnagi.2013.00044

**Published:** 2013-08-23

**Authors:** Jonathon Telianidis, Ya Hui Hung, Stephanie Materia, Sharon La Fontaine

**Affiliations:** ^1^Strategic Research Centre for Molecular and Medical Research, School of Life and Environmental Sciences, Deakin UniversityBurwood, VIC, Australia; ^2^Centre for Cellular and Molecular Biology, School of Life and Environmental Sciences, Deakin UniversityBurwood, VIC, Australia; ^3^Oxidation Biology Unit, Florey Institute of Neuroscience and Mental HealthParkville, VIC, Australia; ^4^Centre for Neuroscience Research, The University of MelbourneParkville, VIC, Australia

**Keywords:** copper, copper homeostasis, ATP7A, ATP7B, Menkes disease, Wilson disease, occipital horn syndrome, ATP7A-related motor neuropathy

## Abstract

Over the past two decades there have been significant advances in our understanding of copper homeostasis and the pathological consequences of copper dysregulation. Cumulative evidence is revealing a complex regulatory network of proteins and pathways that maintain copper homeostasis. The recognition of copper dysregulation as a key pathological feature in prominent neurodegenerative disorders such as Alzheimer’s, Parkinson’s, and prion diseases has led to increased research focus on the mechanisms controlling copper homeostasis in the brain. The copper-transporting P-type ATPases (copper-ATPases), ATP7A and ATP7B, are critical components of the copper regulatory network. Our understanding of the biochemistry and cell biology of these complex proteins has grown significantly since their discovery in 1993. They are large polytopic transmembrane proteins with six copper-binding motifs within the cytoplasmic N-terminal domain, eight transmembrane domains, and highly conserved catalytic domains. These proteins catalyze ATP-dependent copper transport across cell membranes for the metallation of many essential cuproenzymes, as well as for the removal of excess cellular copper to prevent copper toxicity. A key functional aspect of these copper transporters is their copper-responsive trafficking between the *trans-*Golgi network and the cell periphery. ATP7A- and ATP7B-deficiency, due to genetic mutation, underlie the inherited copper transport disorders, Menkes and Wilson diseases, respectively. Their importance in maintaining brain copper homeostasis is underscored by the severe neuropathological deficits in these disorders. Herein we will review and update our current knowledge of these copper transporters in the brain and the central nervous system, their distribution and regulation, their role in normal brain copper homeostasis, and how their absence or dysfunction contributes to disturbances in copper homeostasis and neurodegeneration.

## INTRODUCTION

Copper is indispensable for normal development and function of the central nervous system (CNS). The copper concentration of the human adult brain is significant, estimated at 7–10% of total body copper, similar to that of the liver, the major organ that regulates the copper status of the body (Cartwright and Wintrobe, 1964; Linder, 1991). Regional variance in brain copper concentrations reflects differing metabolic requirements for copper, which change during development (reviewed in Lutsenko et al., 2010). Copper is required as a cofactor for numerous critical enzymes that are involved in vital CNS processes such as respiration, neurotransmitter synthesis, activation of neuropeptides and hormones, protection from oxidative damage, myelination, pigmentation, and iron metabolism among others. The redox cycling of copper between Cu^2^^+^ and Cu^+^ oxidation states is utilized by enzymes involved in these processes for catalytic reactions. However, this redox activity also can lead to elevated reactive oxygen species (ROS) and corruption of critical proteins by adventitious binding of copper ions (Halliwell and Gutteridge, 1984). High oxygen consumption in the brain (20% of total body oxygen), coupled with low levels of antioxidants and antioxidant enzymes, and high levels of metal ions, mean the brain is particularly susceptible to ROS-induced oxidative stress. Hence, precise regulation of brain copper is essential to ensure appropriate levels and distribution for the maintenance of brain function, without risking inadvertent interactions with other cellular components.

Much of what is currently known about brain copper regulation comes from studies of diseases where copper dysregulation is associated with neurodegeneration. Menkes disease (MD; OMIM 309400) is an X-linked inherited disorder with serious neurological symptoms and neurodegeneration resulting from severe copper deficiency. Occipital horn syndrome (OHS; OMIM 304150) is an allelic variant of MD with milder neurological symptoms and predominantly connective tissue defects. In Wilson disease (WD; OMIM 277900), an inherited, autosomal recessive copper toxicosis disorder, patients present with hepatic and neurological symptoms (reviewed in Danks, 1995). There is mounting evidence that copper dysregulation plays a key role in more common neurodegenerative diseases such as Alzheimer’s, Parkinson’s, Huntington’s, motor neuron, and prion diseases (reviewed in Scheiber et al., 2013).

The mechanisms of brain copper import, distribution, and export are now beginning to be elucidated. The exchange of copper between the periphery and the brain is highly regulated by the brain barriers. The copper concentration of cerebrospinal fluid (CSF; ~0.25 μM; Kjellin, 1963; Lentner, 1981) is up to 100-fold lower than that in the plasma (11–25 μM; Tietz, 1987). In a rat brain perfusion study that compared copper uptake into brain capillaries, parenchyma, choroid plexus, and CSF, non-protein bound free copper ion was the predominant copper species that entered the brain via both the blood–brain barrier (BBB) and the blood–CSF barrier (BCB; Choi and Zheng, 2009). The higher rate of copper transport into the brain parenchyma compared to the CSF suggests that the BBB is the main site through which copper enters the brain. Copper influx into the brain parenchyma and CSF is regulated by copper transporters CTR1, ATP7A, and ATP7B, which are highly expressed in the brain capillaries and choroid plexus (Iwase et al., 1996; Qian et al., 1998; Kuo et al., 2006; Niciu et al., 2006; Choi and Zheng, 2009; Donsante et al., 2010; Davies et al., 2013).

**FIGURE 1:**
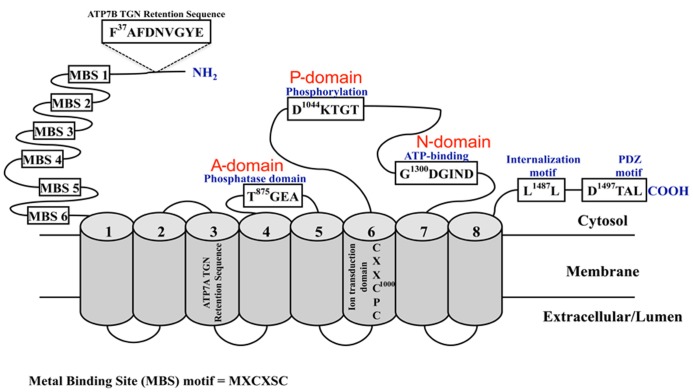
**Schematic diagram of the copper-ATPases, ATP7A and ATP7B.** Shown are the highly conserved domains: the N-terminal copper-binding domain, the phosphatase (A-domain), phosphorylation (P-domain), and ATP-binding (N-domain) domains; and the motifs and sequences required for their localization and trafficking. The cylindrical regions labeled 1 -8 represent the transmembrane domains.

Significant insight into the mechanisms controlling brain copper homeostasis began two decades ago with the identification of the genes encoding the essential copper-transporting ATPases, ATP7A (Chelly et al., 1993; Mercer et al., 1993; Vulpe et al., 1993) and ATP7B (Bull et al., 1993; Petrukhin et al., 1993; Yamaguchi et al., 1993). Mutations in *ATP7A* and *ATP7B* underlie MD and WD, respectively. *ATP7A* is located on chromosome Xq13.2-13.3 and comprises 23 exons that span approximately 150 kb^1^. ATP7B is located on chromosome 13q14.3 and comprises 21 exons that span approximately 80 kb^2^. Transcripts of approximately 7.5–8.5 kb are produced from both genes and contain coding regions of 4.5 kb, which are translated to produce proteins of 180 and 165 kDa, respectively. ATP7A and ATP7B are members of the P_1B_-subfamily of the P-type ATPases. They undergo ATP-dependent cycles of phosphorylation and dephosphorylation to catalyze the translocation of copper across cellular membranes. Their structure and biochemistry was thoroughly reviewed by Lutsenko et al. (2007). They are highly related in structure and function with approximately 60% amino acid identity. They have eight transmembrane domains that form a path through cell membranes for copper translocation; and a large N-terminus with six metal-binding domains (MBDs), each comprising approximately 70 amino acids and the highly conserved metal-binding motif GMxCxxC (where x is any amino acid). Other highly conserved domains include the intramembrane CPC motif that is required for copper translocation through the membrane, the N-domain containing the ATP-binding site, the P-domain containing the conserved aspartic acid residue and the A-domain comprising the phosphatase domain. Copper-binding together with other N- and C-terminal signals regulate their activity, intracellular location, and copper-induced intracellular trafficking (see below and reviewed in La Fontaine and Mercer, 2007; Lutsenko et al., 2007; **Figure 1**).

ATP7A and ATP7B possess dual functions, delivering copper for incorporation into copper-dependent enzymes, and removal of excess copper from cells. These functions are largely regulated by their sub-cellular localization (see below). *ATP7A* is ubiquitously expressed in extrahepatic cells and tissues, which explains the systemic defects caused by its absence or inactivation in MD and points to a house-keeping role for ATP7A. *ATP7B* has a more limited expression pattern, with the highest expression level in the liver, and lower levels in the kidney, placenta, brain, heart, and lungs (Bull et al., 1993; Tanzi et al., 1993; Vulpe et al., 1993). This restricted expression suggests more specialized functions for ATP7B in regulating copper physiology, such as biliary copper excretion (Terada et al., 1999). ATP7B also has a biosynthetic role, supplying copper to cuproenzymes such as ceruloplasmin (Terada et al., 1998). In cells where ATP7B is co-expressed with ATP7A, it often has a specific and distinct role (Veldhuis et al., 2009a; La Fontaine et al., 2010), for example in copper secretion into milk during lactation (Michalczyk et al., 2008), and in fine-tuning the intracellular copper balance in the kidney (Linz et al., 2008; Barnes et al., 2009). The expression of both copper-transporting P-type ATPases (copper-ATPases) in the brain and the severe neurological symptoms that arise from a deficiency of either transporter, suggest that they play key roles in regulating brain copper homeostasis. This review will summarize our current knowledge of the expression, localization, and contribution of ATP7A and ATP7B to maintaining and managing copper levels within the brain.

## EXPRESSION AND LOCALIZATION OF ATP7A AND ATP7B IN THE BRAIN

### ATP7A

**FIGURE 2:**
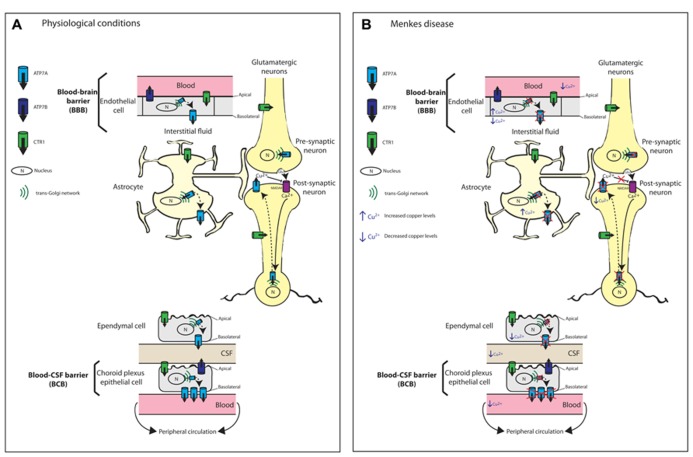
**Schematic diagram illustrating the proposed mechanisms of copper transport across the blood brain barrier (BBB) and blood cerebrospinal fluid barrier (BCB).** **(A)** Copper transport under physiological conditions. Copper import into cells is via the major copper import protein CTR1. The proposed locations and orientation of ATP7A and ATP7B are shown. ATP7A is expressed in cerebrovascular endothelial cells that form the BBB but its expression is 3.4-fold higher in the choroid plexus than in the cerebral capillaries. In both choroid plexus epithelial cells and capillary endothelial cells of the brain, ATP7A predominantly localizes to the basolateral membrane. ATP7A facilitates copper transport from the blood across the BBB to the brain parenchyma. At the choroid plexus, ATP7A facilitates the removal of excess copper from the brain into the blood. Therefore, the BCB serves as the main pathway for eliminating excess brain copper. In contrast, ATP7B is concentrated at the apical membrane. At the CSF-facing apical membrane of choroid plexus epithelial cells, ATP7B may contribute to copper transport into the CSF and copper sequestration in the choroid plexus. Astrocytes are the first brain parenchyma cells to encounter metal ions that cross the BBB and play an important role in copper transport from the blood and CSF toward neurons. Copper transport in glutamatergic neurons is depicted. Copper-independent and reversible trafficking of ATP7A is stimulated by *N*-methyl-D-aspartate receptor (NMDAR) activation that leads to Ca^2+^ influx and is associated with the rapid release of copper from neurons. The released copper down-regulates NMDAR activity thus protecting neurons from excitotoxicity. **(B)** Perturbed copper transport in Menkes disease due to the absence or inactivation of ATP7A. The predicted consequences of ATP7A inactivation in relation to copper levels in brain barrier cells, astrocytes and neurons are shown. Any copper that reaches the BBB accumulates within endothelial cells leading to reduced copper transport to the brain parenchyma, astrocytes and neurons. Any copper that reaches astrocytes also will accumulate. There is reduced copper transport across the BCB. Excitotoxicity due to impaired synaptic copper release from glutamatergic neurons, which down-regulates NMDAR activity, can contribute to seizures and neuronal degeneration in Menkes disease.

The *ATP7A* gene is transcribed to produce an 8.5 kb transcript that is expressed in all tissues examined except for the liver (Chelly et al., 1993; Vulpe et al., 1993). In the mouse brain, the *Atp7a* transcript is expressed in the cerebrovascular endothelial (CVE) cells that form the BBB (Qian et al., 1998), and is strongly expressed in the choroid plexus (Kuo et al., 1997; Murata et al., 1997; Nishihara et al., 1998; Choi and Zheng, 2009), a structure that forms the BCB and regulates the concentration of substances in the CSF (**Figure 2A**). Choi and Zheng (2009) further showed that *Atp7a* is more highly expressed in the brain barriers (BBB and BCB), the brain capillaries and choroid plexus, than in brain parenchyma. Comparing the two barriers, *Atp7a* mRNA expression is 3.4-fold higher in the choroid plexus than in the cerebral capillaries. This observation is consistent with the finding that the Atp7a protein levels in the developing and adult mouse brain are highest in the choroid plexus/ependymal cells of the lateral and third ventricles (Niciu et al., 2006).

Based on abnormal copper accumulation in cultured astrocytes from the macular mouse, a model of MD, Kodama et al. (1991) proposed that astrocytes play an important role in copper transport from the blood and CSF toward neurons, and that this pathway is disturbed in MD and animal models (**Figure 2B**). Kaler and Schwartz (1998) later confirmed the expression of mouse and rat *Atp7a* in astrocytes from various brain regions (cerebral cortex, corpus striatum, and cerebellum). Therefore, ATP7A is postulated to play a key role in the copper distribution from astrocytes to neurons. Importantly, the successful treatment of MD mouse models by intravenous administration of copper earlier than postnatal day 7 (P7; Mann et al., 1979) was proposed to be a consequence of the immaturity of the BBB, which includes astrocytes (Kodama et al., 1991). This immaturity allows the penetration of copper that is prevented in these ATP7A-deficient models once development of the BBB is complete.

Early *in situ* hybridization studies in mouse brain showed significant levels of the *Atp7a* transcript in the hippocampal CA1 region, the dentate gyrus, the cerebellar granular layer and the olfactory bulb, and lower levels in the hippocampal CA3 region and Purkinje neurons (Iwase et al., 1996). *Atp7a* mRNA was detected also by RT-PCR in mouse and rat cerebral cortex and cerebellum, and in isolated rat cerebellar granule neurons (Kaler and Schwartz, 1998). Consistent with these observations, a detailed immunohistochemical study by Niciu et al. (2006) demonstrated the presence of the Atp7a protein in most CNS cell types at P11 in the developing mouse brain and in the adult brain. In transgenic mice that overexpressed human ATP7A, the protein is primarily produced in the CA2 region of the hippocampus, the Purkinje neurons of the cerebellum, and in the choroid plexus (Ke et al., 2006). The overexpression of ATP7A resulted in an overall reduction of brain copper concentrations (Ke et al., 2006), which is consistent with ATP7A in the choroid plexus functioning to efflux copper back into the circulation (Choi and Zheng, 2009). In more recent studies of human brain tissue, ATP7A protein levels are most prominent in the cerebellum and substantia nigra (Davies et al., 2013). The significance of ATP7A expression in these brain regions is poorly understood.

*ATP7A* expression is developmentally regulated (Kuo et al., 1997; Barnes et al., 2005; El Meskini et al., 2005; Niciu et al., 2006). The widespread expression of *Atp7a* mRNA in neurons and ependymal cells during embryonic and postnatal development in the mouse (Kuo et al., 1997; Murata et al., 1997) suggests a house-keeping role for ATP7A in the brain and CNS. Interestingly, *Atp7a* is not detectable by RT-PCR in embryonic day 20 rat astrocytes, but it is detectable in P3, P8, and adult astrocytes (Kaler and Schwartz, 1998). The study by Niciu et al. (2006) showed that Atp7a protein levels are most abundant in the early postnatal period, peaking in the neocortex and cerebellum at P4. From birth (P0) to adulthood, there is a decline in Atp7a levels in most brain regions, and this decline is more pronounced in the hippocampus and cerebellum than in the hypothalamus (Niciu et al., 2006). During this postnatal period, despite a general decline in Atp7a levels, there is increased Atp7a expression in CA2 hippocampal pyramidal cells and cerebellar Purkinje neurons. The authors proposed that since the CA2 region is resistant to epileptogenesis, the increase in CA2 Atp7a levels may contribute to seizure resistance (Niciu et al., 2006). The observed increase in Atp7a levels in Purkinje neurons is consistent with significant levels of ATP7A in human cerebellar Purkinje neurons (Davies et al., 2013), but it is inconsistent with other reports of a postnatal decline in mouse Atp7a mRNA and protein levels in these cells (Iwase et al., 1996; Barnes et al., 2005). Although Barnes et al. (2005) reported a postnatal switch in the expression of Atp7a from Purkinje neurons to Bergmann glia in the rodent brain, in human brain tissue, ATP7A could not be detected in the Bergmann glia (Davies et al., 2013). This discrepancy may allude to the possibility that different mechanisms regulate human and rodent cerebellar copper homeostasis. The increased ATP7A expression in the cerebellum may explain the increased sensitivity of this region, and in particular Purkinje neurons, to copper deficiency as observed in MD (Menkes et al., 1962; Kumode et al., 1993, 1994; Iwase et al., 1996; Geller et al., 1997; Liu et al., 2005a; Niciu et al., 2006).

In the olfactory system, neuronal Atp7a localization correlates with neuronal maturation. Atp7a is initially concentrated in neuronal cell bodies at early embryonic stages, then shifts to the extending axons during the postnatal period (El Meskini et al., 2005). Atp7a levels peak prior to synaptogenesis, which occurs postnatally. Similarly, increased expression levels precede synapse formation following injury-stimulated neurogenesis. Together with a follow-up study of the mottled brindled mouse model of MD (Mo^Br/y^), these data suggest a role for ATP7A in axon extension and synaptogenesis, the absence of which may contribute to the neurodegeneration evident in MD and its mouse models (El Meskini et al., 2007).

The increase in ATP7A during the early postnatal period indicates a crucial role for copper during early development, and particularly in synaptogenesis. This may underlie the critical window during which some human MD patients and mouse models (the mottled mouse mutants) respond favorably to postnatal copper injections, especially when administered in the early postnatal period (Mann et al., 1979; Fujii et al., 1990; Niciu et al., 2006, 2007; Tang et al., 2008). Kaler et al. (2008) reported that this successful copper treatment depends on the presence of a mutant ATP7A protein with some residual copper transport function. Hence, the therapeutic efficacy of copper injections may be a consequence of increased levels of mutant ATP7A, albeit with significantly reduced catalytic activity (La Fontaine et al., 1999; Kaler, 2011).

A zebrafish mutant, *calamity*, with an embryonic-recessive lethal mutation in the *ATP7A* ortholog, shows impaired copper homeostasis with absent melanin pigmentation, defective notochord formation, and neurodegeneration in the hindbrain region of the developing brain (Mendelsohn et al., 2006; Madsen et al., 2008). These authors proposed a developmental hierarchy of copper metabolism, where notochord formation is preferentially preserved during copper limitation, potentially explaining some of the vascular and neurologic abnormalities observed in MD. *Drosophila* has a sole ortholog of *ATP7A*, *DmATP7*, which appears to play an important role in the developing *Drosophila* brain (Norgate et al., 2007). It is strongly expressed in the larval brain at different developmental stages (Burke et al., 2008). Strong *DmATP7A* expression is observed in the ventral ganglion but it is absent from most of the optic lobes (Burke et al., 2008).

### ATP7B

Although ATP7B is expressed in the brain (Bull et al., 1993; Tanzi et al., 1993), its expression patterns and contribution to brain copper homeostasis are less well characterized than that of ATP7A. In the brain of the developing mouse embryo, *Atp7b* mRNA cannot be detected, suggesting that there may be no prenatal expression or that expression is below the detection limits (Kuo et al., 1997). Alternatively, consistent with the possibility that significant expression begins postnatally, brain copper levels in an *Atp7b* null mouse continues to increase slightly throughout adult life (Buiakova et al., 1999). In a study of Atp7b protein and mRNA distribution in the rat brain, Atp7b is detected in the hippocampus, the granular cells of the dentate gyrus and pyramidal cells of the CA1 to CA4 layers, the glomerular cell layer of the olfactory bulbs, Purkinje neurons of the cerebellum, pyramidal neurons of the cerebral cortex, and cores of several nuclei (e.g., pontine nuclei and lateral reticular nuclei) in the brainstem (Saito et al., 1999). In these brain regions, both *Atp7b* mRNA and protein correlate with copper distribution as determined by staining with the copper chelator bathocuproine disulfonic acid (BCS). Based on similar distribution patterns of cuproenzymes such as dopamine-β-hydroxylase (DBH) and Cu–Zn superoxide dismutase (Cu–Zn SOD), as well as abnormal catecholamine synthesis in the Long-Evans Cinnamon (LEC) rat model of WD (Saito et al., 1996; Okabe et al., 1998), these authors speculated that ATP7B-mediated control of copper homeostasis in these brain regions is important in regulating DBH activity.

In contrast to mouse Atp7a, there is continuous Atp7b expression in the adult mouse cerebellar Purkinje neurons, and an age-dependent down-regulation (Barnes et al., 2005). Based on kinetic studies and experiments in mice lacking Atp7b, these authors proposed a homeostatic role for ATP7A in maintaining intracellular copper at a certain level, and a biosynthetic role for ATP7B mediating the synthesis of copper-dependent enzymes such as ceruloplasmin (Barnes et al., 2005).

In the human brain, immunohistochemical staining reveals expression of the ATP7B protein in the visual cortex, anterior cingulate cortex, body of caudate, putamen, substantia nigra, and cerebellum, with the most significant levels of ATP7B detected in the cerebellum, anterior cingulated cortex, and caudate putamen (Davies et al., 2013). Strong staining of ATP7B in Purkinje neurons and not the Bergmann glia is consistent with the findings in the adult rat and mouse brains. However, there is no correlation between ATP7A and ATP7B protein levels and copper levels in the brain regions investigated (Davies et al., 2013).

## FUNCTION AND REGULATION OF THE COPPER-ATPASES IN CNS CELL TYPES

### REGULATION OF ATP7A AND ATP7B

Much of what is currently known about the copper-ATPases derives from studies in peripheral cells and tissues. Emerging studies of copper transport and the copper-ATPases in the brain and certain CNS cell types both support our current understanding of their mechanism of action and regulation, but also provide new insights into the complexity of copper’s role in the CNS.

ATP7A and ATP7B have a dual role in cells; a biosynthetic role, delivering copper to the secretory pathway for metallation of cuproenzymes, and a homeostatic role, exporting excess copper from the cell. Under normal, physiological conditions, ATP7A and ATP7B are localized at the *trans*-Golgi network (TGN) where they provide copper to copper-dependent enzymes synthesized in the secretory pathway. ATP7A has a role in the metallation of enzymes such as peptidylglycine α-amidating monooxygenase (PAM; El Meskini et al., 2003; Steveson et al., 2003), tyrosinase (Petris et al., 2000; Setty et al., 2008), extracellular SOD3 (Qin et al., 2005), DBH (Saito et al., 1996), and lysyl oxidase, all of which are expressed in the CNS (Lutsenko et al., 2010). ATP7A-mediated copper delivery to lysyl oxidase is proposed based on similar temporal expression of these two proteins in the developing rat brain and the sensitivity of lysyl oxidase-dependent processes to ATP7A inactivation as evident in MD and OHS (Royce et al., 1980; Kuivaniemi et al., 1985; Kaler et al., 1994; Kaler, 1998; Tchaparian et al., 2000; Lutsenko et al., 2007). Copper delivery to apo-ceruloplasmin in hepatocytes (Terada et al., 1998) and mouse cerebellum (Barnes et al., 2005) is mediated by Atp7b, and by Atp7a in macrophages in response to hypoxia-mediated increased copper uptake (White et al., 2009).

The key mechanism by which the copper-ATPases regulate cellular copper levels involves alteration of their cellular localization in response to changes in cytoplasmic copper concentration. When intracellular copper levels are elevated, ATP7A and ATP7B traffic from the TGN to the cell periphery to export excess copper from cells. The copper-induced trafficking of ATP7A was first described in Chinese hamster ovary (CHO-K1) cells (Petris et al., 1996), and since then, it has been reported in a wide range of non-polarized (e.g., human fibroblasts, HeLa, and mammary carcinoma cells; Yamaguchi et al., 1996; Ackland et al., 1997; Dierick et al., 1997; Francis et al., 1998; La Fontaine et al., 1998a,b; Qi and Byers, 1998) and polarized cell types (Madin–Darby canine kidney (MDCK), mouse enterocytes, Caco-2; Greenough et al., 2004; Monty et al., 2005; Nyasae et al., 2007). Trafficking of ATP7B also has been observed in non-polarized hepatoma, human bladder carcinoma, and CHO-K1 cells (Hung et al., 1997; Yang et al., 1997; Forbes and Cox, 2000; La Fontaine et al., 2001; Huster et al., 2003), and in polarized hepatic cells (HepG2 and WIF-B; Roelofsen et al., 2000; Guo et al., 2005; Cater et al., 2006).

In polarized cells, ATP7A traffics from the TGN to a rapidly recycling vesicular pool located near the basolateral membrane (Monty et al., 2005; Nyasae et al., 2007), whereas ATP7B traffics to sub-apical vesicles (Roelofsen et al., 2000; Guo et al., 2005; Cater et al., 2006). At these locations they sequester and mediate the export of excess copper. When copper levels return to normal, ATP7A and ATP7B recycle back to the TGN (Petris and Mercer, 1999; Cater et al., 2006; Nyasae et al., 2007). The trafficking and recycling of the copper-ATPases requires specific signal sequences (**Figure 1**). For both ATP7A and ATP7B, only MBD 5 or 6 is necessary and sufficient to mediate copper-stimulated trafficking from the TGN to the cell periphery (Strausak et al., 1999; Cater et al., 2004). C-terminal di- and tri-leucine motifs in ATP7A and ATP7B, respectively, are required for retrograde trafficking of the proteins to the TGN when copper levels return to normal (Petris et al., 1998; Cater et al., 2006; Braiterman et al., 2011), and for basolateral targeting of ATP7A in polarized MDCK cells (Greenough et al., 2004). TGN retention of ATP7A is mediated by a 38 amino acid sequence contained within transmembrane domain three (Francis et al., 1998) whereas ATP7A retention at the basolateral surface may require the PDZ-binding motif (D^1497^TAL) within the C-terminus (Greenough et al., 2004). In ATP7B, a nine amino acid sequence (F^37^AFDNVGYE)**in the N-terminus is essential for TGN retention under low copper conditions and for apical targeting in polarized hepatocytes when copper levels are elevated (Braiterman et al., 2009). Although details of the trafficking machinery involved in these processes remain to be fully elucidated, interacting partners that may play a role include AIPP1, a PDZ domain-containing protein that may bind to the PDZ motif in ATP7A (Stephenson et al., 2005), and dynactin subunit p62 that may be involved in ATP7B trafficking (Lim et al., 2006b).

In addition to the N- and C-terminal signals that control the copper-ATPase response to elevated copper, other processes also contribute to regulating copper-ATPase activity. They include, posttranslational modifications such as transient auto-phosphorylation at the invariant aspartate residue (D^1044^KTGT; **Figure 1**) to form an acyl-phosphate intermediate, a process that is characteristic of the P-type ATPase catalytic cycle (Voskoboinik et al., 1998, 2001a,b; Petris et al., 2002; Tsivkovskii et al., 2002; Cater et al., 2007), copper-stimulated phosphorylation by kinases (Vanderwerf et al., 2001; Voskoboinik et al., 2003; Veldhuis et al., 2009b), and protein interactions. ATOX1 was the first protein shown to interact in a copper-dependent manner with the N-terminal domain of both proteins for delivery of copper to the secretory pathway (Hamza et al., 1999; Larin et al., 1999). MBD 1–4 is most important for this interaction, in particular MBD 2 and 4 (Strausak et al., 2003; Walker et al., 2004; Achila et al., 2006). This is supported by studies in the *Atox1* null mice, which demonstrated a critical requirement for Atox1 in Atp7a function and copper homeostasis (Hamza et al., 2001, 2003). A 45 kDa isoform of the promyelocytic leukemia zinc finger (PLZF) protein, a transcriptional repressor of Hox genes, interacts with the C-terminus of ATP7B (Ko et al., 2006). Both proteins co-localized within the TGN, and based on experiments in HepG2 cells and *Drosophila*, this interaction is proposed to have a role in regulating ERK signal transduction. However, the functional consequences of these associations remain to be elucidated. The glutaredoxin1 (GRX1)/glutathione (GSH) system may play a key role in regulating the trafficking and activity of the copper-ATPases by regulating their redox state through glutathionylation and deglutathionylation by GRX1 (Lim et al., 2006a; Singleton et al., 2010). GRX1 protects proteins from oxidative damage (Mieyal et al., 2008), and it is expressed throughout the brain with highest activity in the hippocampus, cortex and midbrain (Balijepalli et al., 1999; Ehrhart et al., 2002; Aon-Bertolino et al., 2011). Hence, GRX1/GSH may preserve the integrity and function of ATP7A and ATP7B during oxidative stress, which accompanies neuropathological processes. Clusterin (ApoJ) and COMMD1 regulate the degradation of misfolded and mutant copper-ATPases via the lysosomal and proteasomal pathways, respectively (de Bie et al., 2007; Materia et al., 2011, 2012).

### ROLE OF THE COPPER-ATPASES IN CNS CELL TYPES

Information on the activity of the copper-ATPases in specific CNS cell types is limited and somewhat fragmentary, but emerging evidence is revealing specific roles for ATP7A and ATP7B in brain and CNS copper homeostasis. Peripherally, ATP7A and ATP7B mostly have cell type-specific expression patterns and where co-expressed in certain cells and tissues (e.g., kidney, placenta, and mammary gland), they have distinct roles (reviewed in La Fontaine and Mercer, 2007 and Veldhuis et al., 2009a). Similarly in the CNS, ATP7A and ATP7B may be uniquely expressed in certain cell types and co-expressed in others.

#### Choroidal epithelial cells and brain capillary endothelial cells

The BBB and BCB regulate the movement of copper between the brain and peripheral circulation. In both choroid plexus epithelial cells and capillary endothelial cells of the brain, ATP7A predominantly localizes to the basolateral membrane, which is consistent with its location in other polarized epithelial cell types such as intestinal enterocytes (Monty et al., 2005; Nyasae et al., 2007; Davies et al., 2013). In contrast, ATP7B is concentrated at the apical membrane, again consistent with its localization in polarized hepatocytes (Roelofsen et al., 2000; Guo et al., 2005; Cater et al., 2006; Davies et al., 2013). This distinct membrane localization of ATP7A and ATP7B combined with their discrete enzyme kinetics may be a mechanism to ensure strict control over copper transport across the BCB and BBB (Tsivkovskii et al., 2002; Barnes et al., 2005; Hung et al., 2007). The kinetically slower ATP7B at the CSF-facing apical membrane of choroid plexus epithelial cells may explain the slower rate of copper transport into the CSF relative to the choroid plexus copper uptake rate, and may contribute to copper sequestration in the choroid plexus (Barnes et al., 2005, 2009; Choi and Zheng, 2009). In contrast, the basolateral location of the kinetically faster ATP7A facilitates the removal of excess copper from the brain into the blood (Davies et al., 2013). This arrangement of the copper-ATPases is similar to that in other tissues such as the mammary epithelium or the kidney where ATP7A serves to protect from copper excess while ATP7B serves a biosynthetic role (La Fontaine and Mercer, 2007; Veldhuis et al., 2009a). Therefore, the BCB serves as the main pathway for eliminating excess brain copper (**Figure 2**). In a further study that utilized cultured choroidal epithelial Z310 cells, siRNA-mediated knockdown of ATOX1 and ATP7A resulted in increased cellular copper retention, confirming the involvement of ATP7A in copper transport across the choroid plexus and hence in regulating copper homeostasis of the CSF (Monnot et al., 2012). The BBB, which regulates copper influx into the brain, has lower levels of ATP7A at the basolateral surface of brain capillary endothelial cells to enable rapid but limited transport of copper into the brain to accommodate sudden changes in brain copper concentration (Choi and Zheng, 2009).

#### Astrocytes

Astrocytes play a pivotal role in brain copper homeostasis. They are strategically sandwiched between endothelial cells of the BBB and neurons, hence, they are the first brain parenchyma cells to encounter metal ions that cross the BBB. Astrocytes are able to efficiently take up and store copper, and with greater resistance to copper-induced toxicity, they protect neurons from copper toxicity (reviewed in Tiffany-Castiglioni et al., 2011; Scheiber and Dringen, 2012). Atp7a has been detected in rodent astrocytes in culture and in brain tissue (Qian et al., 1997; Barnes et al., 2005; Niciu et al., 2007; Scheiber et al., 2012). These studies implicate Atp7a in copper export from astrocytes, which is consistent with the early observations by Kodama et al. (1991) of copper accumulation in astrocytes of the macular mouse (**Figure 2B**). In astrocytes, similar to peripheral cell types, Atp7a localizes to a perinuclear region and undergoes copper-responsive trafficking between the TGN and the cell periphery. Therefore, it was proposed that Atp7a mediates copper export from astrocytes, which is time-, concentration- and temperature-dependent (Scheiber et al., 2012). However, in contrast to previous studies, copper export from cultured astrocytes is non-saturable and does not follow the established Michaelis–Menten kinetics for ATP7A-dependent copper transport (Scheiber et al., 2012). Therefore, additional ATP7A-independent mechanisms may be involved in copper export from astrocytes. The rate of copper export is proportional to the increase in copper content after copper exposure, which led Scheiber et al. (2012) to propose that the copper export rate is determined by the trafficking or fusion of Atp7a-containing vesicles with the plasma membrane, rather than by ATP7A-mediated transport of copper into vesicles.

#### Neurons

Cerebellar Purkinje neurons are highly sensitive to copper deficiency during development (Lyons and Prohaska, 2009). In the human brain, ATP7A and ATP7B are co-expressed in Purkinje neurons (Davies et al., 2013), and in the mouse brain, for a postnatal period of up to 2 weeks (Barnes et al., 2005). Co-expression of both copper-ATPases may be a mechanism to achieve a delicate balance of copper that is critical to cerebellar development and function. Based on the faster kinetics of copper transport by Atp7a relative to Atp7b, a homeostatic role is proposed for Atp7a in maintaining the intracellular copper level, and a biosynthetic role for Atp7b to mediate the synthesis of copper-dependent enzymes such as ceruloplasmin (Barnes et al., 2005).

Hippocampal ATP7A plays a pivotal role in learning and memory. In mature rat primary hippocampal neurons, Atp7a undergoes both copper-dependent and -independent redistribution from the TGN to dendrites and axons (Schlief et al., 2005). The study by Schlief et al. (2005) provides the first evidence of copper-independent and reversible trafficking of Atp7a that (i) is stimulated by *N*-methyl-D-aspartate (NMDA) receptor activation, (ii) requires Ca^2+^ influx through the NMDA receptor, and (iii) is associated with the rapid release of copper from hippocampal neurons (**Figure 2A**). The inability to detect Atp7a at the cell surface prompted the suggestion that copper may be released by exocytosis from vesicles once Atp7a has recycled back to the TGN. This possibility is consistent with the rapid efflux of copper following NMDA receptor activation. The authors suggest that NMDA receptor activation-mediated trafficking of copper-loaded, ATP7A-associated vesicles provides a readily available and releasable pool of copper following Ca^2^^+^ influx (Schlief et al., 2005). In the hippocampus, following neuronal depolarization, about 15 μM of copper is released into the glutamatergic synaptic cleft from synaptic vesicles (Rajan et al., 1976; Hartter and Barnea, 1988; Kardos et al., 1989; Barnea et al., 1990; Hopt et al., 2003). While the physiological significance of synaptic copper release is not yet clear, it is proposed that copper down-regulates NMDA activity through redox processes that involve *S*-nitrosylation of specific cysteine residues within NMDA subunits NR1 and NR2A, thus protecting hippocampal neurons from excitotoxicity (Schlief et al., 2006). These findings link ATP7A and copper with modulating memory and learning processes that depend on precise regulation of NMDA receptor activity (Schlief et al., 2006). In MD, deficiencies in both brain copper and ATP7A activity will prevent copper release, thus impairing NMDA receptor modulation. The subsequent NMDA-mediated excitotoxicity may contribute to the pathological features, seizures and neuronal degeneration, that are characteristic of MD (Schlief et al., 2006). In Alzheimer’s disease (AD), the combination of increased copper and Aβ [the amyloidogenic cleavage product of the amyloid precursor protein (APP)] in the synaptic cleft may promote the formation of neurotoxic Aβ-copper oligomers contributing to the neuropathology of AD.

#### Motor neurons

The recent identification of a new ATP7A-related distal hereditary motor neuropathy (dHMN), caused by mutations in *ATP7A* (see below), has drawn attention to the role of ATP7A in motor neurons (Kennerson et al., 2010; Yi et al., 2012). The cell bodies of motor neurons are located within the anterior horn of the spinal cord, and their axons extend for long distances to distal limb muscles. Hence, both the CNS and peripheral nervous system are affected in motor neuron disorders (Harding and Thomas, 1980). In undifferentiated NCS-34 cells (a motor neuron-enriched cell line), Atp7a is localized at the TGN and traffics to the cell periphery with elevated copper, as it does in most other cell types. In differentiated NSC-34 cells, Atp7a can be detected along the full length of Tau-1-positive neuritic projections, with localization to the axonal membrane when copper levels are elevated (Yi et al., 2012). Based on the data presented, ATP7A was postulated to traffic along axons, and to mediate copper release from the axonal membrane of motor neurons.

#### Retinal pigment epithelium

Retinal degeneration is observed in both MD and WD (Ferreira et al., 1998). The essential requirement for copper and ATP7B for retinal structure integrity is underscored by reduced thickness of total macula as well as ganglion cell and inner plexiform layer in WD patients. Up to 50% of WD patients have abnormal visual evoked potentials (VEPs; Albrecht et al., 2012). Immunohistochemical studies of mouse and human retina identified the presence of ATP7A in the retinal pigment epithelium (RPE), outer plexiform layer (OPL), and ganglion cell layer (GCL) of the BALB/c mouse retina (Krajacic et al., 2006). In contrast, ATP7B expression is restricted to the RPE. Within the RPE, both copper-ATPases localize to a perinuclear region that overlap with TGN and Golgi markers, consistent with their biosynthetic role in delivering copper to tyrosinase for melanogenesis, and to the iron homeostatic proteins ceruloplasmin and hephaestin. Increased copper levels trigger a redistribution of ATP7B to a diffuse cytoplasmic compartment in an immortalized human RPE cell line.

#### Microglia

Activated microglial cells concentrate at sites of neuronal damage and inflammation. Atp7a expression is elevated in activated microglia surrounding Aβ plaques in the TgCRND8 transgenic mouse model of AD (Zheng et al., 2010). Data from cultured mouse BV-2 microglial cells suggest Atp7a expression and trafficking is responsive to stimulation by the pro-inflammatory cytokine interferon-gamma (IFN-γ). Concomitant copper accumulation due to upregulation of the copper import protein, Ctr1 and copper uptake, suggests a role for copper in the pro-inflammatory pathway. Furthermore, the IFN-γ-stimulated ATP7A redistribution differs from that induced by copper, and appears to mediate copper sequestration in cytoplasmic vesicles rather than copper export. This mechanism is postulated to represent a protective mechanism by activated microglia at sites of amyloidogenesis to reduce aberrant copper-Aβ interactions in the extracellular milieu (Zheng et al., 2010).

#### Pineal gland

Pineal night-specific ATPase (PINA) is a novel splice variant of ATP7B, lacking the N-terminal MBDs and the first four transmembrane domains. It has a predominant nocturnal expression in the pineal gland and retina, under the control of the retina-specific protein, cone rod homeobox (CRX; Li et al., 1998). Despite a large N-terminal truncation, PINA retains a weak copper transport function in a *Saccharomyces cerevisiae ccc2Δ* complementation assay (Borjigin et al., 1999). This finding implicates a yet-to-be-defined role for PINA-mediated copper metabolism in pineal and retinal circadian function (Li et al., 1998; Borjigin et al., 1999).

#### Neuroblastoma cells

Neuroblastoma cells have a high demand for copper for proliferation. High concentrations of copper in neuroblastoma cells are likely achieved via down-regulation of ATP7A expression and thus, reduced copper efflux. Retinoid treatment of neuroblastoma cells causes transcriptional upregulation of *ATP7A* and an increase in ATP7A protein levels (Liu et al., 2005b; Bohlken et al., 2009). Ectopic expression of the retinoic acid receptor *β*_2_ subtype (RAR*β*_2_) also induces ATP7A expression, which is associated a growth inhibitory effect. Conversely, knockdown of ATP7A is associated with reduced copper efflux and increased viability of retinoid-treated cells. These data support a model where malignant neuroblastoma cells have a high copper-dependency for viability and proliferation, and copper depletion by retinoid/RAR*β*_2_-induced ATP7A upregulation offers therapeutic benefit (Bohlken et al., 2009).

## FUNCTIONAL CONTRIBUTION OF ATP7A AND ATP7B TO BRAIN COPPER HOMEOSTASIS

An appreciation of the role of ATP7A and ATP7B in brain copper management derives largely from the functional consequences of their absence or inactivation in the genetically inherited diseases, MD, OHS, WD, and the recently identified ATP7A-related motor neuropathy. The importance of their correct function is also emerging in the context of other neurological diseases where there is perturbed copper metabolism, such as the prion diseases and AD. Our current understanding of the copper-ATPase contribution to brain copper homeostasis and the consequences of their absence or impaired function is summarized below.

### MENKES DISEASE AND OCCIPITAL HORN SYNDROME

The widespread expression of *ATP7A* accounts for the systemic defects that arise from mutation of this gene in MD. This X-linked disease presents in males within the first few months of life, and in severe cases, it is fatal within 2–3 years. The severity of the clinical presentation can vary, but commonly includes abnormal neurodevelopment, seizures associated with cerebral atrophy and dysmyelination, a range of connective tissue and vascular abnormalities, fragile bones, an unusual kinky hair structure (pili torti), hair and skin pigmentation defects and failure to thrive (reviewed in Danks, 1995; Kaler, 2011). These symptoms arise from impaired intestinal copper absorption leading to systemic copper deficiency and consequently, reduced activity of critical copper-dependent enzymes. Paradoxically, copper retention is evident in certain tissues exposed to the limited copper that reaches them and in MD patient skin fibroblast cells (Camakaris et al., 1980; Danks, 1995). Histochemical analysis of brain tissue from affected MD patients revealed neurodegeneration in the cerebral cortex, cerebellum, and hippocampus (Okeda et al., 1991) where ATP7A is enriched (see above).

Occipital horn syndrome is also caused by mutations in *ATP7A*, but it is a milder disease with primarily connective tissue defects and moderate neurological symptoms (Kaler et al., 1994; Kaler, 1998). Causative mutations are often splice site mutations that result in reduced levels of normal *ATP7A* mRNA (reviewed in Kaler, 2011). The milder phenotype suggests that sufficient residual functional ATP7A is produced, but the prominent connective tissue defects indicate that copper delivery to lysyl oxidase is severely disrupted (Kaler, 2011; Scheiber et al., 2013).

The neurological symptoms of MD and OHS have been attributed to impaired ATP7A-mediated copper transport across the BBB (**Figure 2B**), leading to deficiencies of enzymes such as cytochrome *c* oxidase, SOD1, BDH, PAM, lysyl oxidase, and tyrosinase, some of which require ATP7A for metallation in the TGN. The bioactivity of many neuropeptides is dependent on their amidation by PAM, while DBH is important for the production of the neurotransmitter norepinephrine from dopamine (reviewed in Lutsenko et al., 2010; Kaler, 2011). However, impairment of other ATP7A-mediated functions also is likely to contribute to the neurodegeneration in MD. For example, excitotoxicity due to impaired synaptic copper release, which down-regulates NMDA receptor activity, can contribute to seizures and neuronal degeneration (Schlief et al., 2005, 2006; **Figure 2**). In Mo^Br/y^ mice, the absence of a functional ATP7A protein results in degeneration of Purkinje neurons, cytoskeletal abnormalities, and impaired synaptogenesis and axonal outgrowth (Niciu et al., 2006; El Meskini et al., 2007; Niciu et al., 2007).

There are a number of MD mouse models. As noted in the preceding sections, these mouse models have been invaluable in revealing insights into the critical role of ATP7A in the brain. The mottled mice are a series of mouse mutants with mutations in the murine ortholog of *ATP7A.* They exhibit a range of phenotypes that recapitulate the variable clinical severity of the human disease (Levinson et al., 1994; Mercer et al., 1994; Mercer, 1998). The Mo^Br/y^ mouse model resembles most closely classical MD with neurological deficits and early postnatal lethality. This mouse expresses close to normal levels of Atp7a that has severely reduced copper transport activity due to an in-frame deletion of six nucleotides, leading to the loss of highly conserved amino acids A^799^ and L^800^ (Grimes et al., 1997). Studies in this mouse elegantly demonstrated the importance of Atp7a in supplying copper to PAM for the amidation of neuropeptides (Steveson et al., 2003), in axon extension and synaptogenesis, and in compensatory mechanisms to facilitate copper transport across the BBB. The latter includes upregulation of the mutant Atp7a in endothelial cells, as well as increased association of Mo^Br/y^ astrocytes and microglia with the BBB, and these mechanisms possibly contribute to the effectiveness of early copper treatment (El Meskini et al., 2005, 2007; Niciu et al., 2007). The severe copper deficiency in the brain of Mo^Br/y^ mice can be rescued by transgenic expression of the human *ATP7A*. Expression of the *ATP7A* transgene was detected in the cerebellar Purkinje cell layer, the CA1 and CA2 regions of the hippocampus, the mitral layer of the olfactory bulb, the vascular endothelium, and the choroid plexus, but it could not be detected in astrocytes (Llanos et al., 2006). The gene correction significantly improved the survival of the ATP7A-expressing transgenic Mo^Br/y^ mice compared with non-transgenic animals. Furthermore, a recent study demonstrated rescue of neonatal Mo^Br/y^ mice following lateral ventricle injections of adeno-associated virus serotype 5 (AAV5) harboring a truncated *ATP7A* cDNA, in combination with copper treatment (Donsante et al., 2011). The authors proposed different effects of the two treatments: the copper injections increased the amount of brain copper available to levels that were 25–50% of the wild type, while the AAV5 gene delivery improved copper utilization. This was associated with enhanced activity of DBH and correction of brain pathology (Donsante et al., 2011). These findings further illustrate the critical role of copper and ATP7A for brain function and highlight the potential for gene therapy in the treatment of MD patients.

### ATP7A-RELATED MOTOR NEUROPATHY

Recently, missense mutations in *ATP7A* were found to cause a form of dHMN (Kennerson et al., 2010; Yi et al., 2012), which represents the third clinical phenotype associated with mutations in *ATP7A*. dHMNs are a clinically and genetically heterogeneous group of diseases characterized by lower-motor neuron weakness and muscular atrophy (Rossor et al., 2012). Merner et al. (2010) proposed that a common factor in the disease mechanism may be altered copper homeostasis. The phenotype of ATP7A-related motor neuropathy is clinically distinct from that of MD, with variable age of onset that ranges from the first to the sixth decade of life, no overt abnormalities of copper metabolism, and typically distal muscle weakness and atrophy of the lower extremities leading to hand and foot deformities (Kaler, 2011). Nerve conduction studies suggest that the disease causes gradual degeneration of motor neuron axons in the limbs, beginning in the distal portion and progressing toward the cell body (Takata et al., 2004; Kennerson et al., 2009, 2010). The late-onset nature of the disease suggests that these mutations in *ATP7A* have subtle effects on ATP7A function that can take years to manifest clinically.

The missense mutations within *ATP7A* that result in T994I and P1386S substitutions were identified in two families with multiple affected males (Kennerson et al., 2010; Yi et al., 2012). These mutations are outside of the conserved functional domains and they cause abnormal ATP7A trafficking, affecting specifically motor neuron function. An abnormal interaction between ATP7A (T994I) and valosin-containing protein (p97/VCP) was demonstrated, the latter, an ATPase with functions that include vesicular trafficking and protein degradation by the ubiquitin (Ub)-proteasome system (UPS; Watts et al., 2004; Yi et al., 2012). Mutations in p97/VCP are also associated with other diseases that involve motor neuron degeneration (Watts et al., 2004). The significance of this interaction in mediating the disease phenotype awaits further investigation. Potential mechanisms that can lead to axonal degeneration as a result of these *ATP7A* mutations include copper deficiency, oxidative damage from mislocalized copper, and/or slowed synaptic copper release, resulting in inefficient down-regulation of the NMDA receptor and hence excitotoxicity leading to neuronal damage and axonal dieback.

### WILSON DISEASE

In a significant proportion of WD patients, neurodegeneration and neurological presentation reveals an important role for ATP7B in maintaining neuronal copper homeostasis. WD manifests primarily in the liver and brain. Mutations that inactivate ATP7B lead to impaired biliary copper excretion (Terada et al., 1999) and consequently, hepatic copper overload, apoptotic cell death, liver damage, and spillage of copper into the plasma and CSF (Weisner et al., 1987; Kodama et al., 1988; Strand et al., 1998; Gitlin, 2003). Hence, copper also accumulates in extrahepatic tissues, notably the brain, kidneys, and cornea (Danks, 1995; Culotta and Gitlin, 2001).

Approximately 40–50% of WD cases present with neurological symptoms and these patients typically have a later onset than those with the liver disease, presenting in the second or third decade (Das and Ray, 2006; Gouider-Khouja, 2009). Although ATP7B is expressed in several brain regions, brain copper accumulation in WD appears to be secondary to the liver disease, because it can be reversed by transplantation (Emre et al., 2001; Schumacher et al., 2001). The psychiatric symptoms also are reversible with chelation therapy (Madsen and Gitlin, 2007). The main areas of the brain affected in WD patients include the thalamus, subthalamic nuclei, brainstem, cerebellum, and frontal cortex (Das and Ray, 2006). The precise mechanisms mediating neuronal injury in WD are not clear, but potentially involve increased extracellular copper combined with impaired copper homeostasis in those regions of the brain suffering loss of ATP7B function. For example, impaired DBH synthesis may explain the predominant abnormalities of the basal ganglia that result in Parkinsonian symptoms such as rigidity and tremor (Das and Ray, 2006; Pfeiffer, 2007).

The toxic milk mouse (*tx*) and the LEC rat are mouse models of WD that harbor a point mutation (Theophilos et al., 1996) or deletion (Wu et al., 1994), respectively, in *Atp7b*. Studies of the neurological symptoms and neurodegenerative processes in these models have been lacking until recently. One study found that despite an increase in brain copper levels of the *tx* mouse, there are no neurological or behavioral symptoms (Allen et al., 2006). In contrast, a more recent study reported copper deposition in the striatum and hippocampus of *tx* mice associated with an inflammatory response in these tissues, as well as motor and cognitive disturbances and impaired spatial memory (Terwel et al., 2011). The reason for the discrepancy between these two studies is not clear but may relate to the genetic background of the *tx* mice used.

### ALZHEIMER’S DISEASE

Alzheimer’s disease is a progressive neurodegenerative disorder occurring late in life. Patients suffer memory loss and cognitive decline. Key pathological hallmarks include intra- and extracellular proteinaceous deposits (senile plaques comprising the Aβ peptides derived from the processing of APP and neurofibrillary tangles (NFTs) composed of hyperphosphorylated tau). The role of copper in AD is gaining prominence with the discoveries that: (i) it is increased and/or mis-localized in the AD brain; (ii) enriched in extracellular plaques; (iii) deficient in Aβ plaque neighboring brain regions; and (iv) disease-linked proteins, APP, Aβ, tau, and BACE1, are copper-binding proteins with key roles in brain metal regulation (reviewed in Kozlowski et al., 2009; Hung et al., 2010). These observations and findings of dysregulation of other metals such as iron and zinc have led to the “metal theory of AD” with the coining of the term, “metallostasis.” This represents the fatigue of brain metal regulation and distribution, leading to Aβ aggregation and deposition, intraneuronal iron accumulation, and consequently, oxidative injury and neurodegeneration (Roberts et al., 2012; Bush, 2013).

Genetic analysis of AD patients and healthy controls uncovered *ATP7B* as a genetic risk factor for AD. A number of single nucleotide polymorphisms (SNPs) in *ATP7B* are associated with increased risk for AD (Bucossi et al., 2011, 2012; Squitti et al., 2013). These changes occur in either a MBD, the ATP-binding N-domain or transmembrane domains, which may negatively affect ATP7B function in relation to metal-binding, ATP hydrolysis or copper translocation across the membrane. SNPs in the transmembrane domains present the strongest association for AD risk (Squitti et al., 2013). These observations further support a crucial role for ATP7B in maintaining brain copper homeostasis and a potential role in AD pathogenesis. In contrast to these observations, transgenic APP mice (CRND8) that are homozygous for the *tx^J^* mutation, and therefore lacking a functional ATP7B protein, exhibit elevated brain copper levels, but a markedly reduced number of amyloid plaques and decreased plasma Aβ levels (Phinney et al., 2003). The authors postulated that the mechanism of this beneficial effect of the *tx^J^* mutation involves increased clearance of peripheral Aβ pools. Alternatively, elevated intracellular copper due to the *tx^J^* mutation may correct the copper-deficient phenotype of the CRND8 mice. The copper-induced retention of APP at the cell surface, leading to reduced Aβ production and interaction with copper in lipid rafts may explain the decrease in Aβ and amyloid plaques (Hung et al., 2009; Acevedo et al., 2011).

*APOE* and *CLUSTERIN* (*APOJ*) polymorphisms represent the strongest and third strongest genetic risk factors for AD, respectively (Bertram et al., 2007). The encoded proteins have long associations with AD (Mahley et al., 2006; Nuutinen et al., 2009; Yu and Tan, 2012). ApoE and clusterin are well-known for their function as secreted extracellular chaperones with key roles in lipid transport. They have neuroprotective functions, cooperatively regulating the deposition and clearance of Aβ (DeMattos et al., 2004; Wilson et al., 2008). In humans, there are three common *APOE* alleles, *ε2, ε3, *and *ε4*. *APOE-ε4* confers increased risk for AD. The presence of *APOE-ε4* in WD patients with the common H1069Q mutation in *ATP7B* is associated with an earlier onset of symptoms (Litwin et al., 2012). In contrast, *APOE-ε3* is associated with a significant delay in the onset of WD symptoms compared with *APOE-ε4 *carrying patients (Schiefermeier et al., 2000; Wang et al., 2003). This difference may be linked to the isoform-specific antioxidant activity of the ApoE isoforms (Miyata and Smith, 1996). ApoE4 is less effective than ApoE2/ApoE3 as an antioxidant, which may explain the greater susceptibility of ApoE4 patients to copper toxicity. While the exact mechanism remains unclear, these studies suggest a role for ApoE in copper regulation and in influencing WD phenotypes.

We recently demonstrated that clusterin interacts with ATP7A and ATP7B, and this interaction appears to facilitate the degradation of misfolded copper-ATPase molecules predominantly via the lysosome (Materia et al., 2011, 2012). Both MD and WD exhibit a high degree of clinical variability (de Bie et al., 2007; Tumer and Moller, 2009), with reports of identical mutations, even among siblings, conferring variable clinical expression (Duc et al., 1998; Borm et al., 2004). Hence, these observations implicate other factors in determining the clinical phenotype. Based on functional similarities between clusterin and ApoE, the association between ApoE isoforms and WD onset, and the role of clusterin in copper-ATPase degradation, these molecules potentially could play a role in modifying the expression of neurological disease such as AD, MD, and WD. Conceivably, genetic variations in the *clusterin* and *ApoE* alleles, together with environmental factors, could contribute to the variability in the clinical expression of MD and WD.

### PRION DISEASE

Prion diseases are characterized by the continual conformational change of the normal prion protein (PrP^C^) to an infectious, protease-resistant, β-sheet-rich form of the protein (PrP^Sc^; Prusiner, 1982). The resultant toxic PrP^Sc^ aggregates can disrupt axonal transport and synaptic transmission and/or trigger apoptosis, leading to the neurodegenerative pathologies that are collectively termed transmissible spongiform encephalopathies (TSE) (reviewed in Davies and Brown, 2013).

Prion protein is a membrane glycoprotein with four N-terminal octameric repeats and a nearby site that binds copper (Hornshaw et al., 1995a,b; Brown et al., 1997). This protein is ubiquitously expressed but enriched in neurons and concentrated at the synapse (Sales et al., 1998). Its normal physiological role still remains to be fully elucidated but insight into the cell biology and biochemical properties of PrP^C^ is revealing some clues regarding its function. Various studies have demonstrated that copper-binding induces PrP^C^ internalization (Davies and Brown, 2013) prompting the suggestion that PrP^C^ may be involved as a receptor for copper uptake or efflux (Brown and Harris, 2003). More recently, You et al. (2012) confirmed previous findings that copper modulates NMDA receptor activity. They further demonstrated that this occurs through a copper-dependent interaction between PrP^C^ and the GluN1 subunit, which reduces glycine affinity for the receptor, thus suppressing its activity. The role of ATP7A-mediated synaptic copper release may be important in this context but whether it plays a direct role in PrP^C^-copper interactions is not clear.

In contrast to the beneficial role of copper in PrP^C^ function noted above, other studies reported that copper binding to PrP^C^ increases its conversion to PrP^Sc^, and that copper chelation delays the onset of prion disease (Sigurdsson et al., 2003). In support of these studies, disruption of copper homeostasis due to a hypomorphic mutation in *ATP7A* delays the onset of prion disease in mice (Siggs et al., 2012). Copper levels in the brain are reduced by 60% and the amount of the disease-causing PrP^Sc^ is significantly lower than that of the controls. The controversies over the role of copper in PrP^C^ function and prion disease remain to be clarified.

## CONCLUDING STATEMENT

Copper plays a central role in a complex network of signaling pathways that regulate a host of physiological and pathophysiological processes. The crucial role of copper in brain and CNS development and function was highlighted three decades ago when the connection was made between infants with MD and copper deficiency (Danks et al., 1972). However, despite its importance, detailed knowledge of the mechanisms controlling brain copper homeostasis remains limited. Significant advances have been made with the subsequent discovery of key components of the copper regulatory network. The copper-ATPases, ATP7A and ATP7B are among these and since their discovery two decades ago, significant progress has been made toward understanding their pivotal role in normal copper homeostasis. Emerging data now reveal a complex and varied role for copper in the brain, and that the copper-ATPases are integral to the regulation and maintenance of copper-mediated processes within the brain. The consequences of their malfunction are clearly illustrated by the severe neurological deficits and neurodegeneration that accompany the disorders of copper transport, MD and WD. More subtle functional defects in the copper-ATPases or in factors that regulate their activity and/or stability are likely to contribute to other neurodegenerative diseases where copper is dysregulated, such as Alzheimer’s, motor neuron and prion diseases. Given the complexity of the CNS, much remains to be learned about the role of ATP7A and ATP7B in neurological development and neurodegenerative processes. Current ongoing research into the factors that affect the regulation of their expression, post-translational modification and activity will continue to provide new insights into their involvement and adaptive capacity during neuropathological processes associated with aging and disease.

## Conflict of Interest Statement

The authors declare that the research was conducted in the absence of any commercial or financial relationships that could be construed as a potential conflict of interest.

## Footnotes

1 http://asia.ensembl.org/Homo_sapiens/Gene/Summary?g=ENSG00000165240;

2 http://asia.ensembl.org/Homo_sapiens/Gene/Summary?g=ENSG00000123191;

## References

[B1] Acevedo K. M. Hung Y. H. Dalziel A. H. Li Q. X. Laughton K. Wikhe K. (2011). Copper promotes the trafficking of the amyloid precursor protein. *J. Biol. Chem.* 286 8252–8262 10.1074/jbc.M110.128512 21177866 PMC3048711

[B2] Achila D. Banci L. Bertini I. Bunce J. Ciofi-Baffoni S. Huffman D. L. (2006). Structure of human Wilson protein domains 5 and 6 and their interplay with domain 4 and the copper chaperone HAH1 in copper uptake. *Proc. Natl. Acad. Sci. U.S.A.* 103 5729–5734 10.1073/pnas.0504472103 16571664 PMC1458641

[B3] Ackland M. L. Cornish E. J. Paynter J. A. Grimes A. Michalczyk A Mercer J. F. B. (1997). Expression of Menkes disease gene in mammary carcinoma cells. *Biochem. J.* 328 237–243 9359859 10.1042/bj3280237 PMC1218912

[B4] Albrecht P. Müller A.-K. Ringelstein M. Finis D. Geerling G. Cohn E. (2012). Retinal neurodegeneration in Wilson’s disease revealed by spectral domain optical coherence tomography. *PLoS ONE* 7:e49825 10.1371/journal.pone.0049825 PMC3500325 23166778

[B5] Allen K. Buck N. Cheah D. Gazeas S. Bhathal P. Mercer J. (2006). Chronological changes in tissue copper, zinc and iron in the toxic milk mouse and effects of copper loading. *Biometals* 19 555–564 10.1007/s10534-005-5918-5 16937262

[B6] Aon-Bertolino M. L. Romero J. I. Galeano P. Holubiec M. Badorrey M. S. Saraceno G. E. (2011). Thioredoxin and glutaredoxin system proteins-immunolocalization in the rat central nervous system. *Biochim. Biophys. Acta* 1810 93–110 10.1016/j.bbagen.2010.06.011 20620191

[B7] Balijepalli S. Tirumalai P. S. Swamy K. V. Boyd M. R. Mieyal J. J. Ravindranath V. (1999). Rat brain thioltransferase: regional distribution, immunological characterization, and localization by fluorescent in situ hybridization. *J. Neurochem.* 72 1170–1178 10.1046/j.1471-4159.1999.0721170.x 10037490

[B8] Barnea A. Hartter D. Cho G. Bhasker K. Katz B. Edwards M. (1990). Further characterization of the process of in vitro uptake of radiolabeled copper by the rat brain. *J. Inorg. Biochem.* 40 103–110 10.1016/0162-0134(90)80043-W 2092074

[B9] Barnes N. Tsivkovskii R. Tsivkovskaia N. Lutsenko S. (2005). The copper-transporting ATPases, Menkes and Wilson disease proteins, have distinct roles in adult and developing cerebellum. *J. Biol. Chem.* 280 9640–9645 10.1074/jbc.M413840200 15634671

[B10] Barnes N. L. Bartee M. Y. Braiterman L. Gupta A. Ustiyan V. Zuzel V. (2009). Cell-specific trafficking suggests a new role for renal ATP7B in the intracellular copper storage. *Traffic* 10 767–779 10.1111/j.1600-0854.2009.00901.x 19416479 PMC3462735

[B11] Bertram L. Mcqueen M. B. Mullin K. Blacker D. Tanzi R. E. (2007). Systematic meta-analyses of Alzheimer disease genetic association studies: the AlzGene database. *Nat. Genet.* 39 17–23 10.1038/ng1934 17192785

[B12] Bohlken A. Cheung B. B. Bell J. L. Koach J. Smith S. Sekyere E. (2009). ATP7A is a novel target of retinoic acid receptor [beta]2 in neuroblastoma cells. *Br. J. Cancer* 100 96–105 10.1038/sj.bjc.6604833 19127267 PMC2634674

[B13] Borjigin J. Payne A. S. Deng J. Li X. Wang M. W. Ovodenko B. (1999). A novel pineal night-specific ATPase encoded by the Wilson disease gene. *J. Neurosci.* 19 1018–1026 9920665 10.1523/JNEUROSCI.19-03-01018.1999 PMC6782142

[B14] Borm B. Moller L. B. Hausser I. Emeis M. Baerlocher K. Horn N. (2004). Variable clinical expression of an identical mutation in the ATP7A gene for Menkes disease/occipital horn syndrome in three affected males in a single family. *J. Pediatr.* 145 119–121 10.1016/j.jpeds.2004.04.033 15238919

[B15] Braiterman L. Nyasae L. Guo Y. Bustos R. Lutsenko S. Hubbard A. (2009). Apical targeting and Golgi retention signals reside within a 9-amino acid sequence in the copper-ATPase, ATP7B. *Am. J. Physiol. Gastrointest. Liver Physiol.* 296 G433–G444 10.1152/ajpgi.90489.2008 19033537 PMC2643914

[B16] Braiterman L. Nyasae L. Leves F. Hubbard A. L. (2011). Critical roles for the COOH terminus of the Cu-ATPase ATP7B in protein stability, *trans*-Golgi network retention, copper sensing, and retrograde trafficking. *Am. J. Physiol. Gastrointest. Liver Physiol.* 301 G69–G81 10.1152/ajpgi.00038.2011 21454443 PMC3129927

[B17] Brown D. R. Qin K. Herms J. W. Madlung A. Manson J. Strome R. (1997). The cellular prion protein binds copper in vivo. *Nature* 390 684–687 10.1038/37733 9414160

[B18] Brown L. R. Harris D. A. (2003). Copper and zinc cause delivery of the prion protein from the plasma membrane to a subset of early endosomes and the Golgi. *J. Neurochem.* 87 353–363 10.1046/j.1471-4159.2003.01996.x 14511113

[B19] Bucossi S. Mariani S. Ventriglia M. Polimanti R. Gennarelli M. Bonvicini C. (2011). Association between the c. 2495 A>G ATP7B polymorphism and sporadic Alzheimer’s disease. *Int. J. Alzheimers Dis.* 2011 973692 10.4061/2011/973692 PMC3132548 21760992

[B20] Bucossi S. Polimanti R. Mariani S. Ventriglia M. Bonvicini C. Migliore S. (2012). Association of K832R and R952K SNPs of Wilson’s disease gene with Alzheimer’s disease. *J. Alzheimers Dis.* 29 913–919 10.3233/JAD-2012-111997 22356903

[B21] Buiakova O. I. Xu J. Lutsenko S. Zeitlin S. Das K. Das S. (1999). Null mutation of the murine ATP7B (Wilson disease) gene results in intracellular copper accumulation and late-onset hepatic nodular transformation. *Hum. Mol. Genet.* 8 1665–1671 10.1093/hmg/8.9.1665 10441329

[B22] Bull P. C. Thomas G. R. Rommens J. M. Forbes J. R. Cox D. C. (1993). The Wilson disease gene is a putative copper transporting P-type ATPase similar to the Menkes gene. *Nat. Genet.* 5 327–337 (Erratum in *Nat. Genet.* 1994, 6, 214) 10.1038/ng1293-327 8298639

[B23] Burke R. Commons E. Camakaris J. (2008). Expression and localisation of the essential copper transporter DmATP7 in *Drosophila* neuronal and intestinal tissues. *Int. J. Biochem. Cell Biol.* 40 1850–1860 10.1016/j.biocel.2008.01.021 18321764

[B24] Bush A. I. (2013). The metal theory of Alzheimer’s disease. *J. Alzheimers Dis.* 33 S277–S281 10.3233/JAD-2012-129011 22635102

[B25] Camakaris J. Danks D. M. Ackland L. Cartwright E. Borger P Cotton R. G. H. (1980). Altered copper metabolism in cultured cells from human Menkes’ syndrome and mottled mouse mutants. *Biochem. Genet.* 18 117–131 10.1007/BF00504364 7387619

[B26] Cartwright G. E. Wintrobe M. M. (1964). Copper metabolism in normal subjects. *Am. J. Clin. Nutr.* 14 224–232 14142382 10.1093/ajcn/14.4.224

[B27] Cater M. A. Forbes J. La Fontaine S. Cox D Mercer J. F. B. (2004). Intracellular trafficking of the human Wilson protein: the role of the six N-terminal metal binding sites. *Biochem. J.* 380(Pt 3) 805–813 10.1042/BJ20031804 14998371 PMC1224206

[B28] Cater M. A. La Fontaine S. Deal Y. Shield K Mercer J. F. B. (2006). ATP7B mediates vesicular sequestration of copper: insights into biliary copper excretion. *Gastroenterology* 130 493–506 10.1053/j.gastro.2005.10.054 16472602

[B29] Cater M. A. La Fontaine S. Mercer J. F. (2007). Copper binding to the N-terminal metal binding sites or the CPC motif is not essential for copper-induced trafficking of the human Wilson protein (ATP7B). *Biochem. J.* 401 143–153 10.1042/BJ20061055 16939419 PMC1698686

[B30] Chelly J. Tumer Z. Tonnesen T. Petterson A. Ishikawa-Brush Y. Tommerup N. (1993). Isolation of a candidate gene for Menkes disease that encodes a potential heavy metal binding protein. *Nat. Genet.* 3 14–19 10.1038/ng0193-14 8490646

[B31] Choi B. S. Zheng W. (2009). Copper transport to the brain by the blood–brain barrier and blood–CSF barrier. *Brain Res.* 1248 14–21 10.1016/j.brainres.2008.10.056 19014916 PMC2677986

[B32] Culotta V. C. Gitlin J. D. (2001). “Disorders of copper transport,” in *The Metabolic and Molecular Basis of Inherited Disease* 8th Edn eds Scriver C. R. Beaudet A. L. Sly W. S. Valle D. (New York: McGraw-Hill) 3105–3126

[B33] Danks D. M. (1995). “Disorders of copper transport,” in *The Metabolic and Molecular Basis of Inherited Disease* 7th Edn eds Scriver C. R. Beaudet A. L. Sly W. M. Valle D. (New York: McGraw-Hill) 2211–2235

[B34] Danks D. M. Campbell P. E. Stevens B. J. Mayne V. Cartwright E. (1972). Menkes’ kinky hair syndrome. *Pediatrics* 50 188–201 5045349

[B35] Das S. K. Ray K. (2006). Wilson’s disease: an update. *Nat. Clin. Pract. Neurol.* 2 482–493 10.1038/ncpneuro0291 16932613

[B36] Davies K. M. Hare D. J. Cottam V. Chen N. Hilgers L. Halliday G. (2013). Localization of copper and copper transporters in the human brain. *Metallomics* 5 43–51 10.1039/c2mt20151h 23076575

[B37] Davies P. Brown D. R. (2013). “Prion diseases, metals and antioxidants,” in *Brain Diseases and Metalloproteins* ed. Brown D. R. (Boca Raton: Pan Standford Publishing Pte. Ltd.) 249–293

[B38] de Bie P. Muller P. Wijmenga C. Klomp L. W. (2007). Molecular pathogenesis of Wilson and Menkes disease: correlation of mutations with molecular defects and disease phenotypes. *J. Med. Genet.* 44 673–688 10.1136/jmg.2007.052746 17717039 PMC2752173

[B39] de Bie P. Vandesluis B. Burstein E. Berghe P. V. E. V. D. Muller P. Berger R. (2007). Distinct Wilson’s disease mutations in ATP7B are associated with enhanced binding to COMMD1 and reduced stability of ATP7B. *Gastroenterology* 133 1316–1326 10.1053/j.gastro.2007.07.020 17919502 PMC2857755

[B40] DeMattos R. B. Cirrito J. R. Parsadanian M. May P. C. O’Dell M. A. Taylor J. W. (2004). ApoE and clusterin cooperatively suppress A[beta] levels and deposition: evidence that ApoE regulates extracellular A[beta] metabolism in vivo. *Neuron* 41 193–202 10.1016/S0896-6273(03)00850-X 14741101

[B41] Dierick H. A. Adam A. N. Escara-Wilke J. F. Glover T. W. (1997). Immunocytochemical localization of the Menkes copper transport protein (ATP7A) to the *trans* Golgi network. *Hum. Mol. Genet.* 6 409–416 10.1093/hmg/6.3.409 9147644 PMC7185191

[B42] Donsante A. Johnson P. Jansen L. A. Kaler S. G. (2010). Somatic mosaicism in Menkes disease suggests choroid plexus-mediated copper transport to the developing brain. *Am. J. Med. Genet. A* 152A 2529–2534 10.1002/ajmg.a.33632 20799318 PMC3117432

[B43] Donsante A. Yi L. Zerfas P. M. Brinster L. R. Sullivan P. Goldstein D. S. (2011). ATP7A gene addition to the choroid plexus results in long-term rescue of the lethal copper transport defect in a Menkes disease mouse model. *Mol. Ther.* 19 2114–2123 10.1038/mt.2011.143 21878905 PMC3242653

[B44] Duc H. H. Hefter H. Stremmel W. Castaneda-Guillot C. Hernandez Hernandez A. Cox D. W. (1998). His1069Gln and six novel Wilson disease mutations: analysis of relevance for early diagnosis and phenotype. *Eur. J. Hum. Genet.* 6 616–623 9887381 10.1038/sj.ejhg.5200237

[B45] Ehrhart J. Gluck M. Mieyal J. Zeevalk G. D. (2002). Functional glutaredoxin (thioltransferase) activity in rat brain and liver mitochondria. *Parkinsonism Relat. Disord.* 8 395–400 10.1016/S1353-8020(02)00020-2 12217626

[B46] El Meskini R. Cline L. B. Eipper B. A. Ronnett G. V. (2005). The developmentally regulated expression of Menkes protein ATP7A suggests a role in axon extension and synaptogenesis. *Dev. Neurosci.* 27 333–348 10.1159/000086713 16137991

[B47] El Meskini R. Crabtree K. L. Cline L. B. Mains R. E. Eipper B. A. Ronnett G. V. (2007). ATP7A (Menkes protein) functions in axonal targeting and synaptogenesis. *Mol. Cell. Neurosci.* 34 409–421 10.1016/j.mcn.2006.11.018 17215139 PMC1876716

[B48] El Meskini R. Culotta V. C. Mains R. E. Eipper B. A. (2003). Supplying copper to the cuproenzyme peptidylglycine alpha-amidating monooxygenase. *J. Biol. Chem.* 278 12278–12284 10.1074/jbc.M211413200 12529325

[B49] Emre S. Atillasoy E. O. Ozdemir S. Schilsky M. Rathna Varma C. V. Thung S. N. (2001). Orthotopic liver transplantation for Wilson’s disease: a single-center experience. *Transplantation* 72 1232–1236 10.1097/00007890-200110150-00008 11602847

[B50] Ferreira R. Heckenlively J. R. Menkes J. H. Bateman B. (1998). Menkes disease. New ocular and electroretinographic findings. *Ophthalmology* 105 1076–1078 10.1016/S0161-6420(98)96010-9 9627659

[B51] Forbes J. R. Cox D. W. (2000). Copper-dependent trafficking of Wilson disease mutant ATP7B proteins. *Hum. Mol. Genet.* 9 1927–1935 10.1093/hmg/9.13.1927 10942420

[B52] Francis M. J. Jones E. E. Levy E. R. Ponnambalam S. Chelly J. Monaco A. P. (1998). A Golgi localization signal identified in the Menkes recombinant protein. *Hum. Mol. Genet.* 7 1245–1252 10.1093/hmg/7.8.1245 9668166

[B53] Fujii T. Ito M. Tsuda H. Mikawa H. (1990). Biochemical study on the critical period for treatment of the mottled brindled mouse. *J. Neurochem.* 55 885–889 10.1111/j.1471-4159.1990.tb04574.x 2166774

[B54] Geller T. Pan Y. Martin D. (1997). Early neuroradiologic evidence of degeneration in Menkes’ disease. *Pediatr. Neurol.* 17 255–258 10.1016/S0887-8994(97)00092-1 9390704

[B55] Gitlin J. D. (2003). Wilson disease. *Gastroenterology* 125 1868–1877 10.1053/j.gastro.2003.05.010 14724838

[B56] Gouider-Khouja N. (2009). Wilson’s disease. *Parkinsonism Relat. Disord.* 15(Suppl3) S126–S129 10.1016/S1353-8020(09)70798-9 20082972

[B57] Greenough M. Pase L. Voskoboinik I. Petris M. J. O’Brien A. W. Camakaris J. (2004). Signals regulating trafficking of the Menkes (MNK; ATP7A) copper translocating P-type ATPase in polarized MDCK cells. *Am. J. Physiol. Cell Physiol.* 287 C1463–C1471 10.1152/ajpcell.00179.2004 15269005

[B58] Grimes A. Hearn C. J. Lockhart P. Newgreen D. F Mercer J. F. B. (1997). Molecular basis of the brindled mouse mutant (Mobr): a murine model of Menkes disease. *Hum. Mol. Genet.* 6 1037–1042 10.1093/hmg/6.7.1037 9215672

[B59] Guo Y. Nyasae L. Braiterman L. T. Hubbard A. L. (2005). NH2-terminal signals in ATP7B Cu-ATPase mediate its Cu-dependent anterograde traffic in polarized hepatic cells. *Am. J. Physiol. Gastrointest. Liver Physiol.* 289 904–916 10.1152/ajpgi.00262.2005 15994426

[B60] Halliwell B. Gutteridge J. M. (1984). Oxygen toxicity, oxygen radicals, transition metals and disease. *Biochem. J.* 219 1–14 6326753 10.1042/bj2190001 PMC1153442

[B61] Hamza I. Faisst A. Prohaska J. Chen J. Gruss P. Gitlin J. D. (2001). The metallochaperone Atox1 plays a critical role in perinatal copper homeostasis. *Proc. Natl. Acad. Sci. U.S.A.* 98 6848–6852 10.1073/pnas.111058498 11391006 PMC34441

[B62] Hamza I. Prohaska J. Gitlin J. D. (2003). Essential role for Atox1 in the copper-mediated intracellular trafficking of the Menkes ATPase. *Proc. Natl. Acad. Sci. U.S.A.* 100 1215–1220 10.1073/pnas.0336230100 12538877 PMC298753

[B63] Hamza I. Schaefer M. Klomp L. W. J. Gitlin J. (1999). Interaction of the copper chaperone HAH1 with the Wilson disease protein is essential for copper homeostasis. *Proc. Natl. Acad. Sci. U.S.A.* 96 13363–13368 10.1073/pnas.96.23.13363 10557326 PMC23953

[B64] Harding A. E. Thomas P. K. (1980). Genetic aspects of hereditary motor and sensory neuropathy (types I and II). *J. Med. Genet.* 17 329–336 10.1136/jmg.17.5.329 7218272 PMC1048594

[B65] Hartter D. Barnea A. (1988). Evidence for release of copper in the brain: depolarization-induced release of newly taken-up 67copper. *Synapse* 2 412–415 10.1002/syn.890020408 3187909

[B66] Hopt A. Korte S. Fink H. Panne U. Niessner R. Jahn R. (2003). Methods for studying synaptosomal copper release. *J. Neurosci. Methods* 128 159–172 10.1016/S0165-0270(03)00173-0 12948559

[B67] Hornshaw M. P. Mcdermott J. R. Candy J. M. (1995a). Copper binding to the N-terminal tandem repeat regions of mammalian and avian prion protein. *Biochem. Biophys. Res. Commun.* 207 621–629 10.1006/bbrc.1995.1233 7864852

[B68] Hornshaw M. P. Mcdermott J. R. Candy J. M. Lakey J. H. (1995b). Copper binding to the N-terminal tandem repeat region of mammalian and avian prion protein: structural studies using synthetic peptides. *Biochem. Biophys. Res. Commun.* 214 993–999 10.1006/bbrc.1995.2384 7575574

[B69] Hung I. H. Suzuki M. Yamaguchi Y. Yuan D. S. Klausner R. D. Gitlin J. D. (1997). Biochemical characterization of the Wilson disease protein and functional expression in the yeast *Saccharomyces cerevisiae*. *J. Biol. Chem.* 272 21461–21466 10.1074/jbc.272.34.21461 9261163

[B70] Hung Y. H. Bush A. Cherny R. (2010). Copper in the brain and Alzheimer’s disease. *J. Biol. Inorg. Chem.* 15 61–76 10.1007/s00775-009-0600-y 19862561

[B71] Hung Y. H. Layton M. J. Voskoboinik I. Mercer J. F. Camakaris J. (2007). Purification and membrane reconstitution of catalytically active Menkes copper-transporting P-type ATPase (MNK; ATP7A). *Biochem. J.* 401 569–579 10.1042/BJ20060924 17009961 PMC1820817

[B72] Hung Y. H. Robb E. L. Volitakis I. Ho M. Evin G. Li Q. X. (2009). Paradoxical condensation of copper with elevated beta-amyloid in lipid rafts under cellular copper deficiency conditions: implications for Alzheimer disease. *J. Biol. Chem.* 284 21899–21907 10.1074/jbc.M109.019521 19542222 PMC2755914

[B73] Huster D. Hoppert M. Lutsenko S. Zinke J. Lehmann C. Mossner J. (2003). Defective cellular localization of mutant ATP7B in Wilson’s disease patients and hepatoma cell lines. *Gastroenterology* 124 335–345 10.1053/gast.2003.50066 12557139

[B74] Iwase T. Nishimura M. Sugimura H. Igarashi H. Ozawa F. Shinmura K. (1996). Localization of Menkes gene expression in the mouse brain; its association with neurological manifestations in Menkes model mice. *Acta Neuropathol.* 91 482–488 10.1007/s004010050455 8740228

[B75] Kaler S. G. (1998). Metabolic and molecular bases of Menkes disease and occipital horn syndrome. *Pediatr. Dev. Pathol.* 1 85–98 10.1007/s100249900011 10463276

[B76] Kaler S. G. (2011). ATP7A-related copper transport diseases – emerging concepts and future trends. *Nat. Rev. Neurol.* 7 15–29 10.1038/nrneurol.2010.180 21221114 PMC4214867

[B77] Kaler S. G. Gallo L. K. Proud V. K. Percy A. K. Mark Y. Segal N. A. (1994). Occipital horn syndrome and a mild Menkes phenotype associated with splice site mutations at the MNK locus. *Nat. Genet.* 8 195–202 10.1038/ng1094-195 7842019

[B78] Kaler S. G. Holmes C. S. Goldstein D. S. Tang J. Godwin S. C. Donsante A. (2008). Neonatal diagnosis and treatment of Menkes disease. *N. Engl. J. Med.* 358 605–614 10.1056/NEJMoa070613 18256395 PMC3477514

[B79] Kaler S. G. Schwartz J. P. (1998). Expression of the Menkes disease homolog in rodent neuroglial cells. *Neurosci. Res. Commun.* 23 61–66 10.1002/(SICI)1520-6769(199807/08)23:1<61::AID-NRC7>3.0.CO;2-J

[B80] Kardos J. Kovacs I. Hajos F. Kalman M. Simonyi M. (1989). Nerve endings from rat brain tissue release copper upon depolarization. A possible role in regulating neuronal excitability. *Neurosci. Lett.* 103 139–144 10.1016/0304-3940(89)90565-X 2549468

[B81] Ke B.-X. Llanos R. M. Wright M. Deal Y Mercer J. F. B. (2006). Alteration of copper physiology in mice overexpressing the human Menkes protein ATP7A. *Am. J. Physiol. Regul. Integr. Comp. Physiol.* 290 R1460–R1467 10.1152/ajpregu.00806.2005 16397091

[B82] Kennerson M. Nicholson G. Kowalski B. Krajewski K. El-Khechen D. Feely S. (2009). X-linked distal hereditary motor neuropathy maps to the DSMAX locus on chromosome Xq13.1-q21. *Neurology* 72 246–252 10.1212/01.wnl.0000339483.86094.a5 19153371

[B83] Kennerson M. L. Nicholson G. A. Kaler S. G. Kowalski B. Mercer J. F. B. Tang J. (2010). Missense mutations in the copper transporter gene ATP7A cause X-linked distal hereditary motor neuropathy. *Am. J. Hum. Genet.* 86 343–352 10.1016/j.ajhg.2010.01.027 20170900 PMC2833394

[B84] Kjellin K. (1963). Determination of copper in cerebrospinal fluid by activation analysis. *J. Neurochem.* 10 89–93 10.1111/j.1471-4159.1963.tb11468.x 14033253

[B85] Ko J. H. Son W. Bae G. Y. Kang J. H. Oh W. Yoo O. J. (2006). A new hepatocytic isoform of PLZF lacking the BTB domain interacts with ATP7B, the Wilson disease protein, and positively regulates ERK signal transduction. *J. Cell Biochem.* 99 719–734 10.1002/jcb.20980 16676348

[B86] Kodama H. Meguro Y. Abe T. Rayner M. H. Suzuki K. T. Kobayashi S. (1991). Genetic expression of Menkes disease in cultured astrocytes of the macular mouse. *J. Inherit. Metab. Dis.* 14 896–901 10.1007/BF01800470 1779648

[B87] Kodama H. Okabe I. Yanagisawa M. Nomiyama H. Nomiyama K. Nose O. (1988). Does CSF copper level in Wilson disease reflect copper accumulation in the brain? *Pediatr. Neurol.* 4 35–37 10.1016/0887-8994(88)90022-7 3233107

[B88] Kozlowski H. Janicka-Klos A. Brasun J. Gaggelli E. Valensin D. Valensin G. (2009). Copper, iron, and zinc ions homeostasis and their role in neurodegenerative disorders (metal uptake, transport, distribution and regulation). *Coord. Chem. Rev.* 253 2665–2685 10.1016/j.ccr.2009.05.011

[B89] Krajacic P. Qian Y. Hahn P. Dentchev T. Lukinova N. Dunaief J. L. (2006). Retinal localization and copper-dependent relocalization of the Wilson and Menkes disease proteins. *Invest. Ophthalmol. Vis. Sci.* 47 3129–3134 10.1167/iovs.05-1601 16799059

[B90] Kuivaniemi H. Peltonen L. Kivirikko K. I. (1985). Type IX Ehlers-Danlos syndrome and Menkes syndrome: the decrease in lysyl oxidase activity is associated with a corresponding deficiency in the enzyme protein. *Am. J. Hum. Genet.* 37 798–808 9556668 PMC1684617

[B91] Kumode M. Yamano T. Shimada M. (1993). Neuropathological study on cerebellum of macular mutant mouse heterozygote. *Acta Neuropath.* 86 411–417 10.1007/BF00228573 8310789

[B92] Kumode M. Yamano T. Shimada M. (1994). Histochemical study of mitochondrial enzymes in cerebellar cortex of macular mutant mouse, a model of Menkes kinky hair disease. *Acta Neuropath.* 87 313–316 10.1007/BF00296748 8009964

[B93] Kuo Y.-M. Gitschier J. Packman S. (1997). Developmental expression of the mouse mottled and toxic milk genes suggests distinct functions for the Menkes and Wilson disease copper transporters. *Hum. Mol. Genet.* 6 1043–1049 10.1093/hmg/6.7.1043 9215673

[B94] Kuo Y. M. Gybina A. A. Pyatskowit J. W. Gitschier J. Prohaska J. R. (2006). Copper transport protein (Ctr1) levels in mice are tissue specific and dependent on copper status. *J. Nutr.* 136 21–26 16365053 10.1093/jn/136.1.21 PMC2718570

[B95] La Fontaine S. Ackland M. L Mercer J. F. B. (2010). Mammalian copper-transporting P-type ATPases, ATP7A and ATP7B: emerging roles. *Int. J. Biochem. Cell Biol.* 42 206–209 10.1016/j.biocel.2009.11.007 19922814 PMC2846448

[B96] La Fontaine S. Firth S. D. Camakaris J. Englezou A. Theophilos M. B. Petris M. J. (1998a). Correction of the copper transport defect of Menkes patient fibroblasts by expression of the Menkes and Wilson ATPases. *J. Biol. Chem.* 273 31375–31380 10.1074/jbc.273.47.31375 9813047

[B97] La Fontaine S. Firth S. D. Lockhart P. J. Brooks H. Parton R. G. Camakaris J. (1998b). Functional analysis and intracellular localization of the human Menkes protein (MNK) stably expressed from a cDNA construct in Chinese Hamster Ovary cells (CHO-K1). *Hum. Mol. Genet.* 7 1293–1300 10.1093/hmg/7.8.1293 9668172

[B98] La Fontaine S. Firth S. D. Lockhart P. J. Brooks H. Camakaris J Mercer J. F. B. (1999). Intracellular localization and loss of copper-responsiveness of Mnk, the murine homologue of the Menkes protein, in cells from blotchy (Moblo) and brindled (Mobr) mouse mutants. *Hum. Mol. Genet.* 8 1069–1075 10.1093/hmg/8.6.1069 10332039

[B99] La Fontaine S Mercer J. F. B. (2007). Trafficking of the copper-ATPases, ATP7A and ATP7B: role in copper homeostasis. *Arch. Biochem. Biophys.* 463 149–167 10.1016/j.abb.2007.04.021 17531189

[B100] La Fontaine S. Theophilos M. B. Firth S. D. Gould R. Parton R. G Mercer J. F. B. (2001). Effect of the toxic milk mutation (tx) on the function and intracellular localization of Wnd, the murine homologue of the Wilson copper ATPase. *Hum. Mol. Genet.* 10 361–370 10.1093/hmg/10.4.361 11157799

[B101] Larin D. Mekios C. Das K. Ross B. An-Suei Y. Gilliam T. C. (1999). Characterization of the interaction between the Wilson and Menkes disease proteins and the cytoplasmic copper chaperone, HAH1P. *J. Biol. Chem.* 274 28497–28504 10.1074/jbc.274.40.28497 10497213

[B102] Lentner C. (1981). *Geigy Scientific Tables: Units of Measurement, Body Fluids, Composition of the Body, Nutrition*. West Caldwell, NJ: Medical Education Division Ciba-Geigy Corporation

[B103] Levinson B. Vulpe C. Elder B. Martin C. Verley F. Packman S. (1994). The mottled gene is the mouse homologue of the Menkes disease gene. *Nat. Genet.* 6 369–373 10.1038/ng0494-369 8054976

[B104] Li X. Chen S. Wang Q. Zack D. J. Snyder S. H. Borjigin J. (1998). A pineal regulatory element (PIRE) mediates transactivation by the pineal/retina-specific transcription factor CRX. *Proc. Natl. Acad. Sci. U.S.A.* 95 1876–1881 10.1073/pnas.95.4.1876 9465110 PMC19206

[B105] Lim C. M. Cater M. A. Mercer J. F La Fontaine S. (2006a). Copper-dependent interaction of glutaredoxin with the N termini of the copper-ATPases (ATP7A and ATP7B) defective in Menkes and Wilson diseases. *Biochem. Biophys. Res. Commun.* 348 428–436 10.1016/j.bbrc.2006.07.067 16884690

[B106] Lim C. M. Cater M. A. Mercer J. F. B La Fontaine S. (2006b). Copper-dependent interaction of dynactin subunit p62 with the N terminus of ATP7B but not ATP7A. *J. Biol. Chem.* 281 14006–14014 10.1074/jbc.M512745200 16554302

[B107] Linder M. C. (1991). *Biochemistry of Copper*. New York: Plenum Press 10.1007/978-1-4757-9432-8

[B108] Linz R. Barnes N. L. Zimnicka A. M. Kaplan J. H. Eipper B. Lutsenko S. (2008). Intracellular targeting of copper-transporting ATPase ATP7A in a normal and Atp7b -/- kidney. *Am. J. Physiol. Renal. Physiol.* 294 F53–F61 10.1152/ajprenal.00314.2007 17928409

[B109] Litwin T. Gromadzka G. Czlonkowska A. (2012). Apolipoprotein E gene (APOE) genotype in Wilson’s disease: impact on clinical presentation. *Parkinsonism Relat. Disord.* 18 367–369 10.1016/j.parkreldis.2011.12.005 22221592

[B110] Liu P.-C. Chen Y.-W. Centeno J. Quezado M. Lem K. Kaler S. (2005a). Downregulation of myelination, energy, and translational genes in Menkes disease brain. *Mol. Genet. Metab.* 85 291–300 10.1016/j.ymgme.2005.04.007 15923132

[B111] Liu T. Bohlken A. Kuljaca S. Lee M. Nguyen T. Smith S. (2005b). The retinoid anticancer signal: mechanisms of target gene regulation. *Br. J. Cancer* 93 310–318 10.1038/sj.bjc.6602700 16012519 PMC2361573

[B112] Llanos R. M. Ke B.-X. Wright M. Deal Y. Monty F. Kramer D. R. (2006). Correction of a mouse model of Menkes disease by the human Menkes gene. *Biochim. Biophys. Acta* 1762 485–493 10.1016/j.bbadis.2005.12.011 16488577

[B113] Lutsenko S. Barnes N. L. Bartee M. Y. Dmitriev O. Y. (2007). Function and regulation of human copper-transporting ATPases. *Physiol. Rev.* 87 1011–1046 10.1152/physrev.00004.2006 17615395

[B114] Lutsenko S. Bhattacharjee A. Hubbard A. L. (2010). Copper handling machinery of the brain. *Metallomics* 2 596–608 10.1039/c0mt00006j 21072351

[B115] Lyons J. A. Prohaska J. R. (2009). Perinatal copper deficiency alters rat cerebellar purkinje cell size and distribution. *Cerebellum* 9 136–144 10.1007/s12311-009-0136-2 19838760

[B116] Madsen E. Gitlin J. D. (2007). Copper and iron disorders of the brain. *Annu. Rev. Neurosci.* 30 317–337 10.1146/annurev.neuro.30.051606.094232 17367269

[B117] Madsen E. C. Morcos P. A. Mendelsohn B. A. Gitlin J. D. (2008). In vivo correction of a Menkes disease model using antisense oligonucleotides. *Proc. Natl. Acad. Sci. U.S.A.* 105 3909–3914 10.1073/pnas.0710865105 18316734 PMC2268804

[B118] Mahley R. W. Weisgraber K. H. Huang Y. (2006). Apolipoprotein E4: a causative factor and therapeutic target in neuropathology, including Alzheimer’s disease. *Proc. Natl. Acad. Sci. U.S.A.* 103 5644–5651 10.1073/pnas.0600549103 16567625 PMC1414631

[B119] Mann J. R. Camakaris J. Danks D. M. Walliczek E. G. (1979). Copper metabolism in mottled mouse mutants: copper therapy of brindled (Mobr) mice. *Biochem. J.* 180 605–612 573618 10.1042/bj1800605 PMC1161100

[B120] Materia S. Cater M. A. Klomp L. W. Mercer J. F La Fontaine S. (2011). Clusterin (APOJ): a molecular chaperone that facilitates degradation of the copper-ATPases, ATP7A and ATP7B. *J. Biol. Chem.* 286 10073–10083 10.1074/jbc.M110.190546 21242307 PMC3060459

[B121] Materia S. Cater M. A. Klomp L. W. J. Mercer J. F. B La Fontaine S. (2012). Clusterin and COMMD1 independently regulate degradation of the mammalian copper ATPases ATP7A and ATP7B. *J. Biol. Chem.* 287 2485–2499 10.1074/jbc.M111.302216 22130675 PMC3268409

[B122] Mendelsohn B. A. Yin C. Johnson S. L. Wilm T. P. Solnica-Krezel L. Gitlin J. D. (2006). Atp7a determines a hierarchy of copper metabolism essential for notochord development. *Cell Metab.* 4 155–162 10.1016/j.cmet.2006.05.001 16890543

[B123] Menkes J. H. Alter M. Steigleder G. K. Weakley D. R. Sung J. H. (1962). A sex-linked recessive disorder with retardation of growth, peculiar hair, and focal cerebral and cerebellar degeneration. *Pediatrics* 29 764–779 14472668

[B124] Mercer J. F. B. (1998). Menkes syndrome and animal models. *Am. J. Clin. Nutr.* 67(Suppl.) 1022S–1028S 9587146 10.1093/ajcn/67.5.1022S

[B125] Mercer J. F. B. Grimes A. Ambrosini L. Lockhart P. Paynter J. A. Dierick H. (1994). Mutations in the murine homologue of the Menkes disease gene in dappled and blotchy mice. *Nat. Genet.* 6 374–378 10.1038/ng0494-374 8054977

[B126] Mercer J. F. B. Livingston J. Hall B. K. Paynter J. A. Begy C. Chandrasekharappa S. (1993). Isolation of a partial candidate gene for Menkes disease by positional cloning. *Nat. Genet.* 3 20–25 10.1038/ng0193-20 8490647

[B127] Merner N. D. Dion P. A. Rouleau G. A. (2010). Recent advances in the genetics of distal hereditary motor neuropathy give insight to a disease mechanism involving copper homeostasis that may extend to other motor neuron disorders. *Clin. Genet.* 79 23–34 10.1111/j.1399-0004.2010.01591.x 21143467

[B128] Michalczyk A. Bastow E. Greenough M. Camakaris J. Freestone D. Taylor P. (2008). ATP7B expression in human breast epithelial cells is mediated by lactational hormones. *J. Histochem. Cytochem.* 56 389–399 10.1369/jhc.7A7300.2008 18180385 PMC2326107

[B129] Mieyal J. J. Gallogly M. M. Qanungo S. Sabens E. A. Shelton M. D. (2008). Molecular mechanisms and clinical implications of reversible protein S-glutathionylation. *Antioxid. Redox Signal.* 10 1941–1988 10.1089/ars.2008.2089 18774901 PMC2774718

[B130] Miyata M. Smith J. D. (1996). Apolipoprotein E allele-specific antioxidant activity and effects on cytotoxicity by oxidative insults and [beta]-amyloid peptides. *Nat. Genet.* 14 55–61 10.1038/ng0996-55 8782820

[B131] Monnot A. D. Zheng G. Zheng W. (2012). Mechanism of copper transport at the blood-cerebrospinal fluid barrier: influence of iron deficiency in an in vitro model. *Exp. Biol. Med.* (Maywood) 237 327–333 10.1258/ebm.2011.011170 PMC3982225 22442359

[B132] Monty J.-F. Llanos R. M. Mercer J. F. B. Kramer D. R. (2005). Copper exposure induces trafficking of the Menkes protein in intestinal epithelium of ATP7A transgenic mice. *J. Nutr.* 135 2762–2766 16317117 10.1093/jn/135.12.2762

[B133] Murata Y. Kodama H. Abe T. Ishida N. Nishimura M. Levinson B. (1997). Mutation analysis and expression of the mottled gene in the macular mouse model of Menkes disease. *Pediatr. Res.* 42 436–442 10.1203/00006450-199710000-00003 9380433

[B134] Niciu M. J. Ma X. M. El Meskini R. Pachter J. S. Mains R. E. Eipper B. A. (2007). Altered ATP7A expression and other compensatory responses in a murine model of Menkes disease. *Neurobiol. Dis.* 27 278–291 10.1016/j.nbd.2007.05.004 17588765 PMC2040029

[B135] Niciu M. J. Ma X.-M. El-Meskini R. Ronnett G. V. Mains R. E. Eipper B. A. (2006). Developmental changes in the expression of ATP7A during a critical period in postnatal neurodevelopment. *Neuroscience* 139 947–964 10.1016/j.neuroscience.2006.01.044 16549268

[B136] Nishihara E. Furuyama T. Yamashita S. Mori N. (1998). Expression of copper trafficking genes in the mouse brain. *NeuroReport* 9 3259–3263 10.1097/00001756-199810050-00023 9831461

[B137] Norgate M. Southon A. Zou S. Zhan M. Sun Y. Batterham P. Camakaris J. (2007). Copper homeostasis gene discovery in *Drosophila melanogaster*. *Biometals* 20 683–697 10.1007/s10534-006-9075-2 17216353

[B138] Nuutinen T. Suuronen T. Kauppinen A. Salminen A. (2009). Clusterin: a forgotten player in Alzheimer’s disease. *Brain Res. Rev.* 61 89–104 10.1016/j.brainresrev.2009.05.007 19651157

[B139] Nyasae L. Bustos R. Braiterman L. Eipper B. Hubbard A. (2007). Dynamics of endogenous ATP7A (Menkes protein) in intestinal epithelial cells: copper-dependent redistribution between two intracellular sites. *Am. J. Physiol. Gastrointest. Liver Physiol.* 292 G1181–G1194 10.1152/ajpgi.00472.2006 17158254

[B140] Okabe M. Saito S. Saito T. Ito K. Kimura S. Niioka T. (1998). Histochemical localization of superoxide dismutase activity in rat brain. *Free Radic. Biol. Med.* 24 1470–1476 10.1016/S0891-5849(98)00013-6 9641265

[B141] Okeda R. Gei S. Chen I. Okaniwa M. Shinomiya M. Matsubara O. (1991). Menkes’ kinky hair disease: morphological and immunohistochemical comparison of two autopsied patients. *Acta Neuropathol. (Berl.)* 81 450–457 10.1007/BF00293467 2028748

[B142] Petris M. J. Camakaris J. Greenough M. La Fontaine S Mercer J. F. B. (1998). A C-terminal di-leucine is required for localization of the Menkes protein in the *trans*-Golgi network. *Hum. Mol. Genet.* 7 2063–2071 10.1093/hmg/7.13.2063 9817923

[B143] Petris M. J Mercer J. F. B. (1999). The Menkes protein (ATP7A; MNK) cylces via the plasma membrane both in basal and elevated extracellular copper using a C-terminal di-leucine endocytic signal. *Hum. Mol. Genet.* 8 2107–2115 10.1093/hmg/8.11.2107 10484781

[B144] Petris M. J. Mercer J. F. B. Culvenor J. G. Lockhart P. Gleeson P. A. Camakaris J. (1996). Ligand-regulated transport of the Menkes copper P-type ATPase efflux pump from the Golgi apparatus to the plasma membrane: a novel mechanism of regulated trafficking. *EMBO J.* 15 6084–6095 8947031 PMC452430

[B145] Petris M. J. Strausak D Mercer J. F. B. (2000). The Menkes copper transporter is required for the activation of tyrosinase. *Hum. Mol. Genet.* 9 2845–2851 10.1093/hmg/9.19.2845 11092760

[B146] Petris M. J. Voskoboinik I. Cater M. Smith K. Kim B. E. Llanos R. M. (2002). Copper-regulated trafficking of the Menkes disease copper ATPase is associated with formation of a phosphorylated catalytic intermediate. *J. Biol. Chem.* 277 46736–46742 10.1074/jbc.M208864200 12228238

[B147] Petrukhin K. Fischer S. G. Pirastu M. Tanzi R. E. Chernov I. Devoto M. (1993). Mapping, cloning ang genetic characterization of the region containing the Wilson disease gene. *Nat. Genet.* 5 338–343 10.1038/ng1293-338 8298640

[B148] Pfeiffer R. F. (2007). Wilson’s disease. *Semin. Neurol.* 27 123–132 10.1055/s-2007-971173 17390257

[B149] Phinney A. Drisaldi B. Sd Schmidt A. Lugowski S. Coronado V. Liang Y. (2003). In vivo reduction of amyloid-beta by a mutant copper transporter. *Proc. Natl. Acad. Sci. U.S.A.* 100 14193–14198 10.1073/pnas.2332851100 14617772 PMC283568

[B150] Prusiner S. B. (1982). Novel proteinaceous infectious particles cause scrapie. *Science* 216 136–144 10.1126/science.6801762 6801762

[B151] Qi M. Byers P. H. (1998). Constitutive skipping of alternatively spliced exon 10 in the ATP7A gene abolishes Golgi localization of the Menkes protein and produces the occipital horn syndrome. *Hum. Mol. Genet.* 7 465–469 10.1093/hmg/7.3.465 9467005

[B152] Qian Y. Tiffany-Castiglioni E. Harris E. D. (1997). A Menkes P-type ATPase involved in copper homeostasis in the central nervous system of the rat. *Brain Res. Mol. Brain Res.* 48 60–66 10.1016/S0169-328X(97)00083-1 9379850

[B153] Qian Y. Tiffany-Castiglioni E. Welsh J. Harris E. D. (1998). Copper efflux from murine microvascular cells requires expression of the Menkes disease Cu-ATPase. *J. Nutr.* 128 1276–1282 9687544 10.1093/jn/128.8.1276

[B154] Qin Z. Itoh S. Jeney V. Ushio-Fukai M. Fukai T. (2005). Essential role for the Menkes ATPase in activation of extracellular superoxide dismutase: implication for vascular oxidative stress. *FASEB J.* 334–336 10.1096/fj.05-4564fje 16371425

[B155] Rajan K. Colburn R. Davis J. (1976). Distribution of metal ions in the subcellular fractions of several rat brain areas. *Life Sci.* 18 423–431 10.1016/0024-3205(76)90220-4 1256247

[B156] Roberts B. R. Ryan T. M. Bush A. I. Masters C. L. Duce J. A. (2012). The role of metallobiology and amyloid-β peptides in Alzheimer’s disease. *J. Neurochem.* 120 149–166 10.1111/j.1471-4159.2011.07500.x 22121980

[B157] Roelofsen H. Wolters H. Luyn M. J. A. V. Miura N. Kuipers F. Vonk R. J. (2000). Copper-induced apical trafficking of ATP7B in polarized hepatoma cells provides a mechanism for biliary copper excretion. *Gastroenterology* 119 782–793 10.1053/gast.2000.17834 10982773

[B158] Rossor A. M. Kalmar B. Greensmith L. Reilly M. M. (2012). The distal hereditary motor neuropathies. *J. Neurol. Neurosurg. Psychiatry* 83 6–14 10.1136/jnnp-2011-300952 22028385

[B159] Royce P. M. Camakaris J. Danks D. M. (1980). Reduced lysyl oxidase activity in skin fibroblasts from patients with Menkes’ syndrome. *Biochem. J.* 192 579–586 6112984 10.1042/bj1920579 PMC1162373

[B160] Saito T. Nagao T. Okabe M. Saito K. (1996). Neurochemical and histochemical evidence for an abnormal catecholamine metabolism in the cerebral cortex of the Long-Evans Cinnamon rat before excessive copper accumulation in the brain. *Neurosci. Lett.* 216 195–198 8897491 10.1016/0304-3940(96)13041-x

[B161] Saito T. Okabe M. Hosokawa T. Kurasaki M. Hata A. Endo F. (1999). Immunohistochemical determination of the Wilson copper-transporting P-type ATPase in the brain tissues of the rat. *Neurosci. Lett.* 266 13–16 10.1016/S0304-3940(99)00258-X 10336172

[B162] Sales N. Rodolfo K. Hassig R. Faucheux B. Di Giamberardino L. Moya K. L. (1998). Cellular prion protein localization in rodent and primate brain. *Eur. J. Neurosci.* 10 2464–2471 10.1046/j.1460-9568.1998.00258.x 9749773

[B163] Scheiber I. Dringen R Mercer J. F. B. (2013). “Copper: effects of deficiency and overload,” in *Interrelations between Essential Metal Ions and Human Diseases* eds Sigel A. Sigel H. Sigel R. K. O. (Dordrecht: Springer) (in press) 10.1007/978-94-007-7500-8_11 24470097

[B164] Scheiber I. F. Dringen R. (2012). Astrocyte functions in the copper homeostasis of the brain. *Neurochem. Int.* 62 556–565 10.1016/j.neuint.2012.08.017 22982300

[B165] Scheiber I. F. Schmidt M. M. Dringen R. (2012). Copper export from cultured astrocytes. *Neurochem. Int.* 60 292–300 10.1016/j.neuint.2011.12.012 22226844

[B166] Schiefermeier M. Kollegger H. Madl C. Polli C. Oder W. Kuhn H. (2000). The impact of apolipoprotein E genotypes on age at onset of symptoms and phenotypic expression in Wilson’s disease. *Brain* 123(Pt 3) 585–590 10.1093/brain/123.3.585 10686180

[B167] Schlief M. L. Craig A. M. Gitlin J. D. (2005). NMDA receptor activation mediates copper homeostasis in hippocampal neurons. *J. Neurosci.* 25 239–246 10.1523/JNEUROSCI.3699-04.2005 15634787 PMC6725203

[B168] Schlief M. L. West T. Craig A. M. Holtzman D. M. Gitlin J. D. (2006). Role of the Menkes copper-transporting ATPase in NMDA receptor-mediated neuronal toxicity. *Proc. Natl. Acad. Sci. U.S.A.* 103 14919–14924 10.1073/pnas.0605390103 17003121 PMC1578502

[B169] Schumacher G. Platz K. P. Mueller A. R. Neuhaus R. Luck W. Langrehr J. M. (2001). Liver transplantation in neurologic Wilson’s disease. *Transplant. Proc.* 33 1518–1519 10.1016/S0041-1345(00)02578-1 11267403

[B170] Setty S. R. G. Tenza D. Sviderskaya E. V. Bennett D. C. Raposo G. Marks M. S. (2008). Cell-specific ATP7A transport sustains copper-dependent tyrosinase activity in melanosomes. *Nature* 454 1142–1146 10.1038/nature07163 18650808 PMC2812007

[B171] Siggs O. M. Cruite J. T. Du X. Rutschmann S. Masliah E. Beutler B. (2012). Disruption of copper homeostasis due to a mutation of Atp7a delays the onset of prion disease. *Proc. Natl. Acad. Sci. U.S.A.* 109 13733–13738 10.1073/pnas.1211499109 22869751 PMC3427069

[B172] Sigurdsson E. M. Brown D. R. Alim M. A. Scholtzova H. Carp R. Meeker H. C. (2003). Copper chelation delays the onset of prion disease. *J. Biol. Chem.* 278 46199–46202 10.1074/jbc.C300303200 14519758

[B173] Singleton W. C. Mcinnes K. T. Cater M. A. Winnall W. R. Mckirdy R. Yu Y. (2010). Role of glutaredoxin1 and glutathione in regulating the activity of the copper-transporting P-type ATPases, ATP7A and ATP7B. *J. Biol. Chem.* 285 27111–27121 10.1074/jbc.M110.154468 20566629 PMC2930710

[B174] Squitti R. Polimanti R. Bucossi S. Ventriglia M. Mariani S. Manfellotto D. (2013). Linkage disequilibrium and haplotype analysis of the ATP7B gene in Alzheimer’s disease. *Rejuvenation Res.* 16 3–10 10.1089/rej.2012.1357 22950421 PMC3582274

[B175] Stephenson S. E. M. Dubach D. Lim C. M. Mercer J. F. B La Fontaine S. (2005). A single PDZ domain protein interacts with the Menkes copper ATPase, ATP7A: a new protein implicated in copper homeostasis. *J. Biol. Chem.* 280 33270–33279 10.1074/jbc.M505889200 16051599

[B176] Steveson T. C. Ciccotosto D. D. Ma X.-M. Mueller G. P. Mains R. E. Eipper B. A. (2003). Menkes protein contirbutes to the function of peptidylglycine a-amidating monooxygenase. *Endocrinology* 144 188–200 10.1210/en.2002-220716 12488345

[B177] Strand S. Hofmann W. J. Grambihler A. Hug H. Volkmann M. Otto G. (1998). Hepatic failure and liver cell damage in acute Wilson’s disease involve CD95 (APO-1/Fas) mediated apoptosis. *Nat. Med.* 4 588–593 10.1038/nm0598-588 9585233

[B178] Strausak D. Howie M. K. Firth S. D. Schlicksupp A. Pipkorn R. Multhaup G. (2003). Kinetic analysis of the interaction of the copper chaperone Atox1 with the metal binding sites of the Menkes protein. *J. Biol. Chem.* 278 20821–20827 10.1074/jbc.M212437200 12679332

[B179] Strausak D. La Fontaine S. Hill J. Firth S. D. Lockhart P. J Mercer J. F. B. (1999). The role of GMXCXXC metal binding sites in the copper-induced redistribution of the Menkes protein. *J. Biol. Chem.* 274 11170–11177 10.1074/jbc.274.16.11170 10196202

[B180] Takata R. I. Speck Martins C. E. Passosbueno M. R. Abe K. T. Nishimura A. L. Da Silva M. D. (2004). A new locus for recessive distal spinal muscular atrophy at Xq13.1-q21. *J. Med. Genet.* 41 224–229 10.1136/jmg.2003.013201 14985388 PMC1735691

[B181] Tang J. Donsante A. Desai V. Patronas N. Kaler S. G. (2008). Clinical outcomes in Menkes disease patients with a copper-responsive ATP7A mutation, G727R. *Mol. Genet. Metab.* 95 174–181 10.1016/j.ymgme.2008.06.015 18752978 PMC2654537

[B182] Tanzi R. E. Petrukhin K. Chernov I. Pellequer J. L. Wasco W. Ross B. (1993). The Wilson disease gene is a copper transporting ATPase with homology to the Menkes disease gene. *Nat. Genet.* 5 344–350 10.1038/ng1293-344 8298641

[B183] Tchaparian E. H. Uriu-Adams J. Y. Keen C. L. Mitchell A. E. Rucker R. B. (2000). Lysyl oxidase and P-ATPase-7A expression during embryonic development in the rat. *Arch. Biochem. Biophys.* 379 71–77 10.1006/abbi.2000.1842 10864443

[B184] Terada K. Aiba N. Yang X.-L. Iida M. Nakai M. Miura N. (1999). Biliary excretion of copper in LEC rat after introduction of copper transporting P-type ATPase, ATP7B. *FEBS Lett.* 448 53–56 10.1016/S0014-5793(99)00319-1 10217409

[B185] Terada K. Nakako T. Yang X.-L. Iida M. Aiba N. Minamiya Y. (1998). Restoration of holoceruloplasmin synthesis in LEC rat after infusion of recombinant adenovirus bearing WND cDNA. *J. Biol. Chem.* 273 1815–1820 10.1074/jbc.273.3.1815 9430732

[B186] Terwel D. Loschmann Y. N. Schmidt H. H. Scholer H. R. Cantz T. Heneka M. T. (2011). Neuroinflammatory and behavioural changes in the Atp7B mutant mouse model of Wilson’s disease. *J. Neurochem.* 118 105–112 10.1111/j.1471-4159.2011.07278.x 21517843

[B187] Theophilos M. B. Cox D. W Mercer J. F. B. (1996). The toxic milk mouse is a murine model of Wilson disease. *Hum. Mol. Genet.* 5 1619–1624 10.1093/hmg/5.10.1619 8894697

[B188] Tietz N. W. (1987). *Fundamentals of Clinical Chemistry*. Philadelphia: Saunders

[B189] Tiffany-Castiglioni E. Hong S. Qian Y. (2011). Copper handling by astrocytes: insights into neurodegenerative diseases. *Int. J. Dev. Neurosci.* 29 811–818 10.1016/j.ijdevneu.2011.09.004 21968186

[B190] Tsivkovskii R. Eisses J. F. Kaplan J. H. Lutsenko S. (2002). Functional properties of the copper-transporting ATPase ATP7B (the Wilson’s disease protein) expressed in insect cells. *J. Biol. Chem.* 277 976–983 10.1074/jbc.M109368200 11677246

[B191] Tumer Z. Moller L. B. (2009). Menkes disease. *Eur. J. Hum. Genet.* 18 511–518 10.1038/ejhg.2009.187 19888294 PMC2987322

[B192] Vanderwerf S. M. Cooper M. J. Stetsenko I. V. Lutsenko S. (2001). Copper specifically regulates intracellular phosphorylation of the Wilson’s disease protein, a human copper-transporting ATPase. *J. Biol. Chem.* 276 36289–36294 10.1074/jbc.M102055200 11470780

[B193] Veldhuis N. Gaeth A. Pearson R. Gabriel K. Camakaris J. (2009a). The multi-layered regulation of copper translocating P-type ATPases. *Biometals* 22 177–190 10.1007/s10534-008-9183-2 19130269

[B194] Veldhuis N. A. Valova V. A. Gaeth A. P. Palstra N. Hannan K. M. Michell B. J. (2009b). Phosphorylation regulates copper-responsive trafficking of the Menkes copper transporting P-type ATPase. *Int. J. Biochem. Cell Biol.* 41 2403–2412 10.1016/j.biocel.2009.06.008 19576997

[B195] Voskoboinik I. Brooks H. Smith S. Shen P. Camakaris J. (1998). ATP-dependent copper transport by the Menkes protein in membrane vesicles isolated from cultured Chinese hamster ovary cells. *FEBS Lett.* 438 178–182 10.1016/S0014-5793(98)01059-X 9762903

[B196] Voskoboinik I. Fernando R. Veldhuis N. Hannan K. M. Marmy-Conus N. Pearson R. B. (2003). Protein kinase-dependent phosphorylation of the Menkes copper P-type ATPase. *Biochem. Biophys. Res. Commun.* 303 337–342 10.1016/S0006-291X(03)00329-2 12646208

[B197] Voskoboinik I. Greenough M. La Fontaine S. Mercer J. F. B. Camakaris J. (2001a). Functional studies on the Wilson copper P-type ATPase and toxic milk mouse mutant. *Biochem. Biophys. Res. Comm.* 281 966–970 10.1006/bbrc.2001.4445 11237756

[B198] Voskoboinik I. Mar J. Strausak D. Camakaris J. (2001b). The regulation of catalytic activity of the menkes copper-translocating P-type ATPase. Role of high affinity copper-binding sites. *J. Biol. Chem.* 276 28620–28627 10.1074/jbc.M103532200 11373292

[B199] Vulpe C. Levinson B. Whitney S. Packman S. Gitschier J. (1993). Isolation of a candidate gene for Menkes disease and evidence that it encodes a copper-transporting ATPase. *Nat. Genet.* 3 7–13 10.1038/ng0193-7 8490659

[B200] Walker J. M. Huster D. Ralle M. Morgan C. T. Blackburn N. J. Lutsenko S. (2004). The N-terminal metal-binding site 2 of the Wilson’s Disease Protein plays a key role in the transfer of copper from Atox1. *J. Biol. Chem.* 279 15376–15384 10.1074/jbc.M400053200 14754885

[B201] Wang X. P. Wang X. H. Bao Y. C. Zhou J. N. (2003). Apolipoprotein E genotypes in Chinese patients with Wilson’s disease. *QJM* 96 541–542 10.1093/qjmed/hcg093 12881597

[B202] Watts G. D. Wymer J. Kovach M. J. Mehta S. G. Mumm S. Darvish D. (2004). Inclusion body myopathy associated with Paget disease of bone and frontotemporal dementia is caused by mutant valosin-containing protein. *Nat. Genet.* 36 377–381 10.1038/ng1332 15034582

[B203] Weisner B. Hartard C. Dieu C. (1987). CSF copper concentration: a new parameter for diagnosis and monitoring therapy of Wilson’s disease with cerebral manifestation. *J. Neurol. Sci.* 79 229–237 10.1016/0022-510X(87)90275-9 3612170

[B204] White C. Kambe T. Fulcher Y. G. Sachdev S. W. Bush A. I. Fritsche K. (2009). Copper transport into the secretory pathway is regulated by oxygen in macrophages. *J. Cell Sci.* 122 1315–1321 10.1242/jcs.043216 19351718 PMC2671928

[B205] Wilson M. R. Yerbury J. J. Poon S. (2008). Potential roles of abundant extracellular chaperones in the control of amyloid formation and toxicity. *Mol. Biosyst.* 4 42–52 10.1039/b712728f 18075673

[B206] Wu J. Forbes J. R. Chen H. S. Cox D. W. (1994). The LEC rat has a deletion in the copper transporting ATPase gene homologous to the Wilson disease gene. *Nat. Genet.* 7 541–544 10.1038/ng0894-541 7951327

[B207] Yamaguchi Y. Heiny M. E. Gitlin J. D. (1993). Isolation and characterization of a human liver cDNA as a candidate gene for Wilson disease. *Biochem. Biophys. Res. Comm.* 197 271–277 10.1006/bbrc.1993.2471 8250934

[B208] Yamaguchi Y. Heiny M. E. Suzuki M. Gitlin J. D. (1996). Biochemical characterization and intracellular localization of the Menkes disease protein. *Proc. Natl. Acad. Sci. U.S.A.* 93 14030–14035 10.1073/pnas.93.24.14030 8943055 PMC19489

[B209] Yang X.-L. Miura N. Kawarada Y. Terada K. Petrukhin K. Gilliam T. C. (1997). Two forms of Wilson disease protein produced by alternative splicing are localized in distinct cellular compartments. *Biochem. J.* 326 897–902 9307043 10.1042/bj3260897 PMC1218748

[B210] Yi L. Donsante A. Kennerson M. L. Mercer J. F. Garbern J. Y. Kaler S. G. (2012). Altered intracellular localization and valosin-containing protein (p97 VCP) interaction underlie ATP7A-related distal motor neuropathy. *Hum. Mol. Genet.* 21 1794–1807 10.1093/hmg/ddr612 22210628 PMC3313796

[B211] You H. Tsutsui S. Hameed S. Kannanayakal T. J. Chen L. Xia P. (2012). Abeta neurotoxicity depends on interactions between copper ions, prion protein, and *N*-methyl-D-aspartate receptors. *Proc. Natl. Acad. Sci. U.S.A.* 109 1737–1742 10.1073/pnas.1110789109 22307640 PMC3277185

[B212] Yu J. T. Tan L. (2012). The role of clusterin in Alzheimer’s disease: pathways, pathogenesis, and therapy. *Mol. Neurobiol.* 45 314–326 10.1007/s12035-012-8237-1 22274961

[B213] Zheng Z. White C. Lee J. Peterson T. S. Bush A. I. Sun G. Y. (2010). Altered microglial copper homeostasis in a mouse model of Alzheimer’s disease. *J. Neurochem.* 114 1630–1638 10.1111/j.1471-4159.2010.06888.x 20626553 PMC2945454

